# Multi-kingdom profiling reveals altered gut phage-bacteria-metabolite interactions in MASLD

**DOI:** 10.1038/s41467-026-71981-0

**Published:** 2026-04-18

**Authors:** Xiaofeng Zhou, Da Zhou, Yanni Pu, Hanseul Kim, Zhonghan Sun, Wenhao Qi, Jiadong Jin, Wanqin Zhang, Mingfeng Xia, Chengyan Wang, Shangyu Hong, Long H. Nguyen, Na Jiao, Yan Zheng, Taotao Liu

**Affiliations:** 1 Ministry of Education Key Laboratory of Contemporary Anthropology, Human Phenome Institute, Fudan University, Shanghai, China; 2 State Key Laboratory of Genetics and Development of Complex Phenotypes, School of Life Science, Fudan University, Shanghai, China; 3 Department of Gastroenterology and Hepatology, Zhongshan Hospital of Fudan University, Shanghai, China; 4 Shanghai Institute of Liver Disease, Shanghai, China; 5 Department of Biostatistics, Harvard T.H. Chan School of Public Health, Boston, MA USA; 6 Clinical and Translational Epidemiology Unit, Massachusetts General Hospital and Harvard Medical School, Boston, MA USA; 7 Division of Gastroenterology, Massachusetts General Hospital and Harvard Medical School, Boston, MA USA; 8 Department of Endocrinology and Metabolism, Zhongshan Hospital and Fudan Institute for Metabolic Diseases, Fudan University, Shanghai, China; 9 Human Phenome Institute, Fudan University, Shanghai, China

**Keywords:** Gastroenterology, Microbiology

## Abstract

Metabolic dysfunction-associated steatotic liver disease (MASLD) is increasingly linked to gut microbial dysbiosis, but most studies have focused on bacteria, neglecting viruses and fungi, and their interactions. Here we show that MASLD is characterized by coordinated disruption of bacterial, viral and fungal communities and by a disturbed phage-bacteria-metabolite axis associated with disease-related bile acid changes. Integrating shotgun metagenomics, fungal ITS2 sequencing, fecal metabolomics and clinical profiling in 210 patients with MASLD and 210 age- and gender-matched healthy controls, we find reduced microbial diversity and extensive remodeling of cross-kingdom ecological networks in MASLD. *Ruminococcus gnavus* emerges as an enriched central hub, while *Faecalibacterium prausnitzii* and its associated bacteriophages are depleted. Phage-host analyses further reveal reduced lytic activity against *R. gnavus* and loss of sulfur amino acid metabolism-related auxiliary metabolic genes, which may impair *F. prausnitzii* fitness. Diminished phage control may facilitate *R. gnavus* expansion, alongside increased fecal isodeoxycholic acid, a secondary bile acid implicated in hepatic steatosis. A diagnostic classifier integrating bacterial and viral features with clinical parameters distinguish MASLD from controls in our cohort and maintain predictive performance in two external datasets. Together, these findings uncover a disrupted phage-bacteria-metabolite axis in MASLD and provide a multi-kingdom framework for non-invasive biomarker discovery and microbiome-targeted therapies.

## Abstract (web summary)

Here, using multi-omics, the authors show that MASLD is associated with alterations in gut bacteria, viruses, and fungi, and identify potential disruptions in phage-bacteria-metabolite interactions, highlighting microbial signatures and ecological changes linked to disease.

## Introduction

Metabolic dysfunction-associated steatotic liver disease (MASLD) has emerged as a leading global cause of liver-related morbidity and mortality^1^, affecting approximately 30% of the population worldwide^2^. Despite its growing burden, mechanistic understanding and effective therapeutical options for MASLD remain limited.

Recent advances in microbiome research have implicated gut microbial dysbiosis as a key contributor to MASLD pathogenesis^3–6^. Microbial metabolites mediate host-microbiota interactions affecting lipid metabolism, inflammation, and fibrosis^7,8^. However, most studies have focused on bacterial taxa, often overlooking the broader microbial ecosystem, including fungi^9^ and viruses^10^. Notably, bacteriophages, i.e., viruses that infect bacteria, play a central role in shaping gut microbial dynamics and have shown relevance in metabolic disorders^11^. For example, lytic bacteriophages can attenuate pathogenic bacteria reducing cytolysin levels in alcoholic hepatitis^12^, and fecal virome transplantation has demonstrated efficacy in modulating bacterial composition and metabolic traits in type 2 diabetes^13^. Nevertheless, the contribution of virus–bacteria interactions in MASLD remains poorly characterized. In addition, gut microbial signatures also hold promise as non-invasive biomarkers, offering an attractive alternative to liver biopsy in MASLD. Yet, most existing microbiome studies are limited by sample sizes, and insufficient adjustment for confounders, potentially leading to biased associations^14^.

In this work, we perform a comprehensive, multi-omics analysis in a well-matched cohort of 210 patients with MASLD and 210 healthy controls. By integrating bacterial, viral (bacteriophage), and fungal profiling with fecal metabolomics and clinical assessments, we characterize multi-kingdom dysbiosis and disrupted phage-bacteria interactions in MASLD. We further investigate how these alterations are associated with disease-related metabolic changes and develop a microbiome-based classifier for non-invasive disease detection that remains predictive across external datasets.

## Results

### Participant’s characteristics and study design

**Fig. 1:**
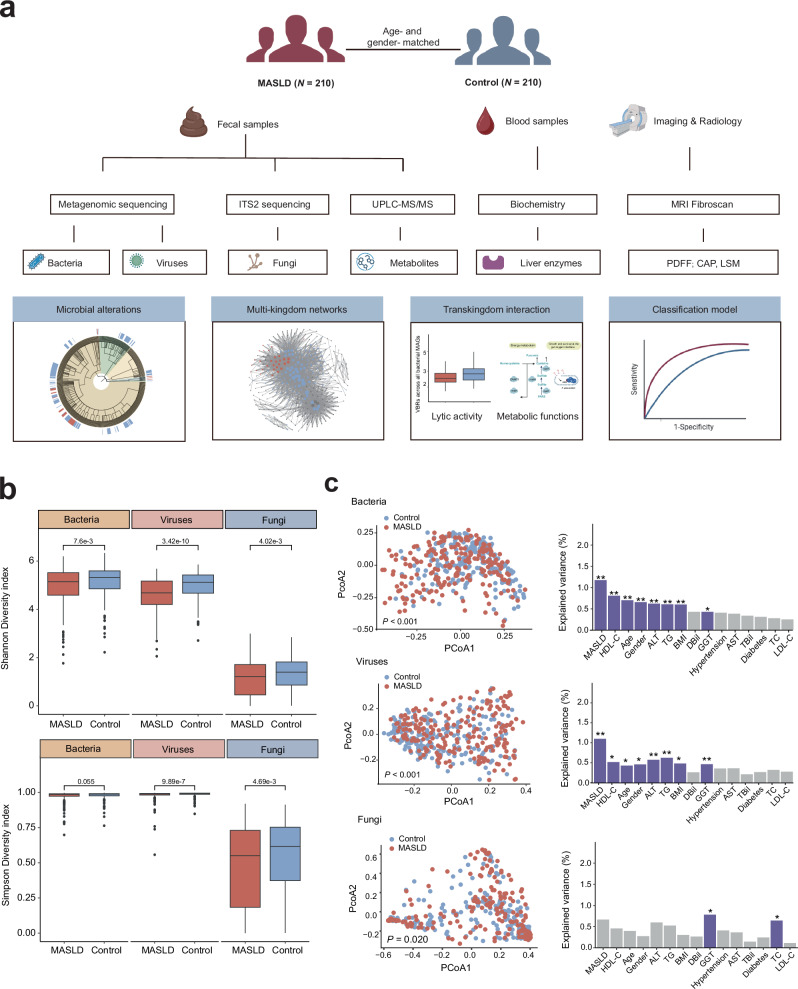
Study design and altered gut microbial structures in MASLD patients. **a** Schematic overview of the study design (icons created in BioRender. Zhou, X. (2026) https://BioRender.com/2cgs072). 210 patients with MASLD and 210 age- and gender-matched healthy controls are enrolled. Multi-omics profiling is performed to comprehensively characterize the gut ecosystem, including metagenomics and ITS2 sequencing for the bacterial, viral and fungal community analysis, targeted fecal metabolomics, and clinical assessments of liver function and disease severity. **b** Alpha diversity, measured by Shannon and Simpson indices, across bacterial, viral, and fungal communities in patients with MASLD (red; *n* = 210individuals) and healthy controls (blue; *n* = 210individuals) (two-sided Wilcoxon rank-sum test). Box plots show the median, interquartile range, and 1.5 × IQR; outliers are shown as points. Each data point represents one independent participant. **c** Variations in gut microbial community structure explained by host factors. Left: Principal coordinates analysis (PCoA) based on Bray–Curtis dissimilarity, with samples colored by disease group (healthy controls, blue; MASLD, red; *P* < 0.001, FDR = 0.008 for bacteria, *P* < 0.001, FDR = 0.008 for viruses and *P* = 0.02, FDR = 0.06 for fungi). Right: Proportion of variation in gut microbial composition explained by individual host factors, estimated by PERMANOVA with Benjamini–Hochberg correction. Purple bars indicate FDR < 0.05 and grey bars indicate FDR ≥ 0.05; *FDR < 0.05, **FDR < 0.01, ***FDR < 0.001, ****FDR < 0.0001. PDFF proton density fat fraction, CAP controlled attenuation parameter, LSM liver stiffness measurement, ALT alanine aminotransferase, AST aspartate aminotransferase, GGT gamma-glutamyl transferase, DBil direct bilirubin, TBil total bilirubin, TC total cholesterol, TG triglycerides.

To comprehensively characterize the multi-kingdom microbial signatures associated with MASLD, we established an age- and gender-matched case-control study involving 420 participants recruited from Zhongshan Hospital in Shanghai between 2021 and 2022 (Fig. 1a). MASLD was diagnosed primarily through clinical evaluations and abdominal ultrasonography. Participants were excluded if they reported significant alcohol consumption (≥20 g per day for male and ≥10 g per day for female), had a diagnosis of cancer or serious diseases affecting major organs (except MASLD in case participants), had undergone major surgery within the previous 8 weeks, or had used antibiotics within the prior 4 weeks (see detailed inclusion and exclusion criteria in “Methods”). Among the enrolled participants, 30.5% were female, with a median age of 40 years (Supplementary Data 1). Clinical parameters, including liver function indicators, were consistent with established diagnostic criteria for MASLD, and medication use was minimal (metformin, *n* = 2; proton pump inhibitors, *n* = 5) (Supplementary Data 1).

Fecal samples from all enrolled participants underwent multi-omics profiling to characterize MASLD-associated alterations in the gut ecosystem and metabolome, yielding metagenomic (*n* = 420), fungal ITS2 sequencing (*n* = 413), and fecal metabolomic (*n* = 414) datasets. These datasets were integrated with clinical metadata to identify microbial signatures associated with MASLD.

### Distinct gut microbiome compositions in MASLD

**Fig. 2:**
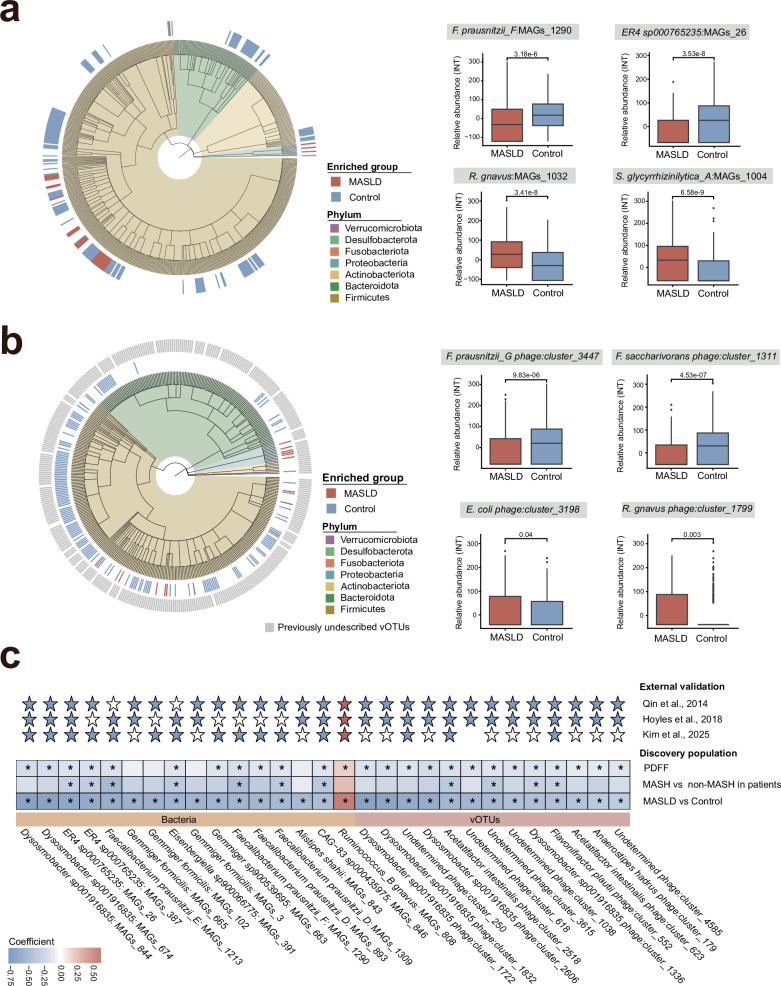
Gut bacterial and viral signatures associated with MASLD. Cladogram of **a** bacterial metagenome-assembled genomes (MAGs) and **b** viral operational taxonomic units (vOTUs; only those with predicted bacterial hosts shown) show microbiomes significantly altered between MASLD patients (*n* = 210 individuals) and healthy controls (*n* = 210 individuals). For both trees, phyla, including predicted host phyla for vOTUs, are indicated by the different background shading colors of the tree sectors. Outer ring denotes differential microbial signatures: taxa positively associated with MASLD are marked in red, while those negatively associated are marked in blue, respectively (linear regression, FDR < 0.05). An additional ring in (**b**) indicates whether the vOTUs described before. Boxplots on the right of (**a**, **b**) illustrate examples of microbial features that were significantly altered in patients with MASLD (data were inverse-normal transformed, the central band represents the median, the hinges represent the first and third quartiles, the whiskers represent the 1.5× interquartile range from the hinge, and the dots represent the outliers). **c** Associations of MASLD-related bacterial MAGs (orange) and vOTUs (light pink) with MASLD severity-related traits. Only features validated in at least two external cohorts are shown. Replication across independent cohorts is indicated by stars. Red and blue denote positive and negative associations, respectively, between microbial features and MASLD, hepatic steatosis, or cirrhosis. Significance is defined as FDR < 0.05 for the Qin et al. and Kim et al. cohorts, and *P* < 0.05 for the Hoyles et al. cohort due to limited sample size). White stars indicate non-significant associations. Microbial features validated in at least two cohorts are further assessed for associations with proton density fat fraction (PDFF), MASH status and MASLD in the discovery cohort using linear regression adjusted for age, gender, and BMI (*FDR < 0.05 for PDFF and MASLD, *P* < 0.05 for MASH vs. non-MASH).

We profiled the gut bacteriome and virome using shotgun metagenomic sequencing with rigorous annotation pipelines incorporating co-binning strategies. A total of 1344 non-redundant bacterial metagenome-assembled genomes (MAGs) and 5440 dereplicated viral operational taxonomic units (vOTUs) were constructed. Most bacterial MAGs were classified within Firmicutes, followed by Bacteroidota (Supplementary Fig. 1a). Of note, a few bacterial MAGs were annotated as putative new species, primarily within the *Collinsella* genus based on integrated analysis using GTDB-tk^15^ and PhyloPhlAn^16^ (see details in “Methods”). Virome analysis where species-level clustering was performed referencing the Unified Human Gut Virome Catalog (UHGV), revealed substantial viral diversity, with 67.94% (3696/5440) of vOTUs previously uncharacterized (Fig. 2b). The dominant viral families were *Siphoviridae* (71.03%) and *Myoviridae* (13.43%) (Supplementary Fig. 1b). Using CRISPR spacer matching and sequence similarity^17^, we predicted 16,250 virus-bacterial host linkages, involving 58.47% of vOTUs and 94.20% of bacterial MAGs. We found that although a moderate number of MAGs were constructed for *F. prausnitzii* in this study (12 MAGs; Supplementary Data 2), a relatively large number of phages were predicted to infect this species. This pattern may reflect the high abundance of *F. prausnitzii* in the gut microbiome and pronounced inter-individual strain specificity, and have been previously reported^18,19^. ITS2 sequencing identified 439 fungal genera, dominated by *Saccharomyces*, *Aspergillus* and *Candida* (Supplementary Fig. 1c).

Patients with MASLD showed marked reductions in α-diversity across all three microbial kingdoms, as measured by Shannon and Simpson indices (all *P* < 0.01, Fig. 1b). Microbial community structure was influenced by MASLD status and host factors (all *P* < 0.05), with MASLD status explaining the largest variance in bacterial and viral compositions, followed by age, gender, BMI, and metabolic indicators. In contrast, fungal community was less associated with MASLD, with host factors playing a more prominent role (Fig. 1c).

### Comprehensive microbial taxonomic alterations associated with MASLD and its progression

To explore MASLD-associated changes in gut microbiota, we analyzed prevalent microbial features at their respective taxonomic resolutions, defined as features detected in at least 10% of individuals with average relative abundance ≥0.01%. Specifically, bacterial features were represented by MAGs, viral features by vOTUs, and fungal features at the genus level, resulting in 626 bacterial MAGs, 585 viral vOTUs, and 64 fungal genera. Using multivariable linear regression with adjustment for age, gender, and BMI, we identified 173 (27.6%) bacterial MAGs, 243 (41.5%) vOTUs, and 9 (14.1%) fungal genera significantly associated with MASLD (FDR < 0.05; Fig. 2a, b, Supplementary Fig. 2a and Supplementary Data 3–5). MASLD patients exhibited higher abundances of MAGs belonging to *Schaedlerella glycyrrhizinilytica* (for example, *S. glycyrrhizinilytica_A: MAGs_1004*), *Escherichia coli* (*E. coli: MAGs_1201*), and *Ruminococcus gnavus* (*R. gnavus: MAGs_1032*), and a marked depletion of *Faecalibacterium prausnitzii* (for example, *F. prausnitzii: MAGs_1290*), a well-established anti-inflammatory and hepatoprotective bacterium^20–22^ (Fig. 2a). Notably, an unclassified bacterial MAG potentially representing a putative new *Collinsella* species was also significantly reduced in MASLD patients (Supplementary Data 3), suggesting the possible role of previously undescribed taxa in disease pathophysiology. Virome analysis revealed parallel shifts, including depletion of phages targeting *F. prausnitzii* and *Fusicatenibacter saccharivorans*, and enrichment of those infecting *E. coli* and *R. gnavus* in MASLD patients (Fig. 2b and Supplementary Data 4). Mycobiome profiling further identified altered abundances of several fungal genera, notably a reduction of *Penicillium* and an enrichment of *Alternaria*, the latter of which has been previously associated with lipid metabolism^23^ (Supplementary Fig. 2 and Supplementary Data 5).

To further assess clinical relevance, we analyzed associations between these microbial signatures with MASLD severity, including liver fat content measured by magnetic resonance imaging-proton density fat fraction (MRI-PDFF, *n* = 366), and FibroScan Controlled Attenuation Parameter (CAP) scores in patients with MASLD (*n* = 172), as well as fibrosis severity assessed by FibroScan Liver Stiffness Measurement (LSM) scores in the same patients (*n* = 172). Beyond these indicators, at-risk metabolic dysfunction-associated steatohepatitis (MASH) was defined among MASLD patients using a FibroScan-AST (FAST) score ≥0.35 (*n* = 88)^24,25^. In addition, we validated these signatures in three independent replication datasets: (1) Dataset 1, consisting of 98 cirrhosis patients and 83 healthy controls, in which cirrhosis represents the terminal stage of fibrotic progression (Qin et al.)^26^; (2) Dataset 2, comprising of 56 MASLD patients, including 10 with stage 0–1 steatosis and the remainder with more advanced steatosis (Hoyles et al.)^27^; and (3) Dataset 3, including 211 MASLD patients and 502 controls from a cohort of female Caucasian participants (Kim et al.)^28^.

Among the 173 MASLD-associated bacterial MAGs, 16 exhibited statistically significant and directionally consistent associations across at least two independent external replication datasets. Similarly, 13 MASLD-associated vOTUs were replicated in at least two external datasets (FDR < 0.05 for the cirrhosis and MASLD replication dataset, *P* < 0.05 for the steatosis dataset, Fig. 2c and Supplementary Data 6). These microbial features further showed a consistent pattern across disease progression, showing concordant alterations in MASH patients compared with non-MASH MASLD patients and significant association with liver fat content measured by PDFF (Fig. 2c). For example, *R. gnavus: MAGs_808* which was enriched in MASLD patients in our discovery cohort, was consistently enriched in patients with cirrhosis, severe steatosis, and independent MASLD cohorts, and showed a positive association with liver fat content. In contrast, *F. prausnitzii: MAGs_1213*, which was depleted in MASLD patients and replicated in external steatosis and MASLD datasets, showed further depletion in MASH patients and was negatively associated with liver fat content. Fungal associations were less pronounced, with *Penicillium* abundance inversely associated with hepatic fat content (Supplementary Fig. 2b). Together, these findings underscore extensive multi-kingdom microbial dysbiosis in MASLD, characterized by both taxonomic and disease stage-related microbial alterations, suggesting their potential roles in disease pathogenesis and progression.

### Distinct intra- and inter-kingdom correlation networks in MASLD

**Fig. 3:**
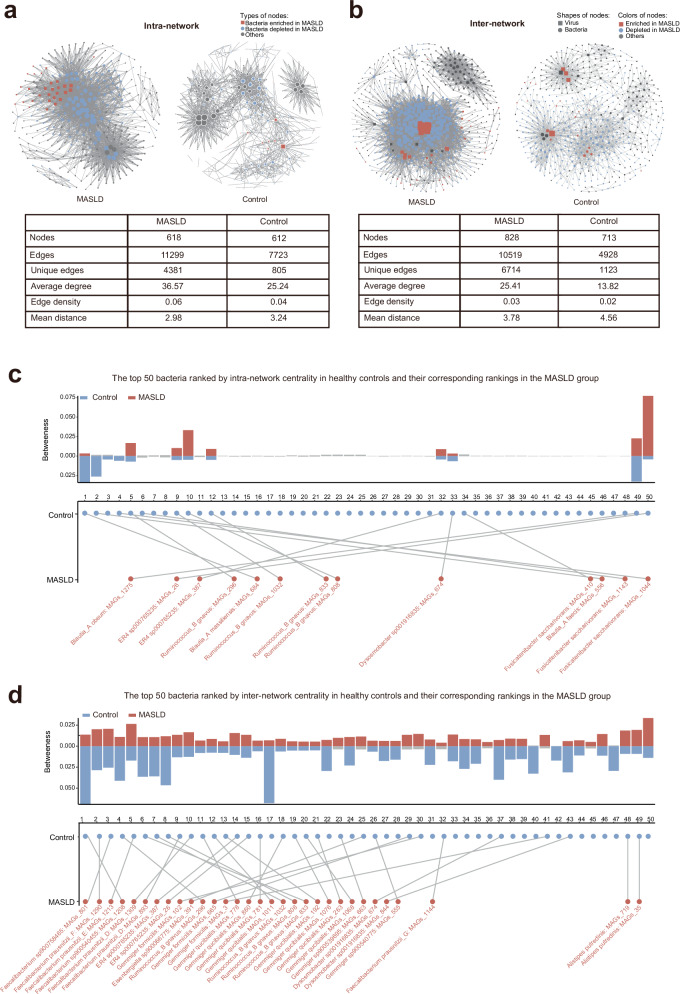
Altered intra- and inter-kingdom correlation networks in MASLD. **a**, **b** Partial Spearman correlation depicting **a** bacterial intra-kingdom correlation networks, and **b** inter-kingdom networks, along with their corresponding topological characteristics in MASLD patients (left) and healthy controls (right), adjusting for age, gender and BMI. Nodes are colored according to the group in which they are enriched, and node size reflects network degree. In the inter-kingdom networks, node shapes indicate microbial kingdom (circles represent bacteria and squares represent viruses). **c**, **d** The top 50 hub nodes identified in the (**c**) intra-kingdom networks and the inter-kingdom networks (**d**). Of these, 13 and 31 bacterial taxa, respectively, retain their hub status in the corresponding MASLD networks. For both panels, node betweenness centralities are illustrated in bar plots, with red bars indicating values in the MASLD network and blue bars indicating control network. Node with betweenness centralities below the group mean are shown in grey.

Despite the observed reduction in α-diversity across all microbial kingdoms in MASLD (Fig. 1b), intra-kingdom microbial networks exhibited greater topological complexity compared to those in healthy controls. This was reflected by higher average degree, greater edge density, and reduced mean shortest path distance, indicating more densely interconnected community structures. These patterns were consistently observed across bacterial (Fig. 3a), viral, and fungal networks (Supplementary Fig. 3a, b). MASLD-specific networks contained substantially more unique correlations than those of controls: 4381 vs. 805 (bacteria), 4312 vs. 668 (viruses), and 79 vs. 39 (fungi), respectively (*P* < 0.05, Fig. 3a and Supplementary Fig. 3c, d), suggesting widespread rewiring of microbial interactions in disease. Of note, within viral networks, vOTUs enriched in healthy controls displayed significantly higher connectivity, reflected by elevated node degrees, than those nondifferential, or enriched in MASLD (Supplementary Fig. 3f), a pattern not observed in bacterial or fungal networks (Supplementary Fig. 3e, g).

Inter-kingdom network analysis further revealed that MASLD patients exhibited markedly more complex cross-domain interactions than controls (Fig. 3b), with a sixfold increase in unique correlations (6714 in MASLD patients vs. 1123 in controls). These inter-kingdom networks also showed higher average node degree, denser connectivity, and shorter mean path lengths, reinforcing a globally perturbed microbial ecosystem in MASLD.

To characterize how disease status influenced key microbial nodes, we identified hub taxa based on degree rankings. Within intra-kingdom bacterial networks, only 13 MAGs retained hub status across both MASLD and control groups, reflecting extensive rewiring (Fig. 3c). *R. gnavus* MAGs exhibited elevated betweenness centrality in MASLD, highlighting their enhanced role in mediating microbial interactions, despite slightly lower degree rankings. In contrast, *Fusicatenibacter saccharivorans* MAGs showed reduced centrality and degree in MASLD, indicating loss of ecological importance.

In inter-kingdom networks, hub taxa were somewhat more stable, with 31 of the top 50 bacterial MAGs maintaining hub status across both groups (Fig. 3d), potentially reflecting conserved phage-bacteria co-abundance relationships. Of note, microbial nodes enriched in healthy controls often remained central to network architecture even in MASLD patients (Supplementary Fig. 3h), underscoring their foundational role in maintaining gut ecosystem stability. In particular, all four *R. gnavus* MAGs consistently emerged as central hubs in both intra- and inter-kingdom networks and exhibited high betweenness centrality (Fig. 3c, d), suggesting their critical role in microbial community restructuring and metabolic disturbances associated with MASLD.

### Bacteriophage–bacteria interactions shape gut microbial architecture in MASLD

**Fig. 4:**
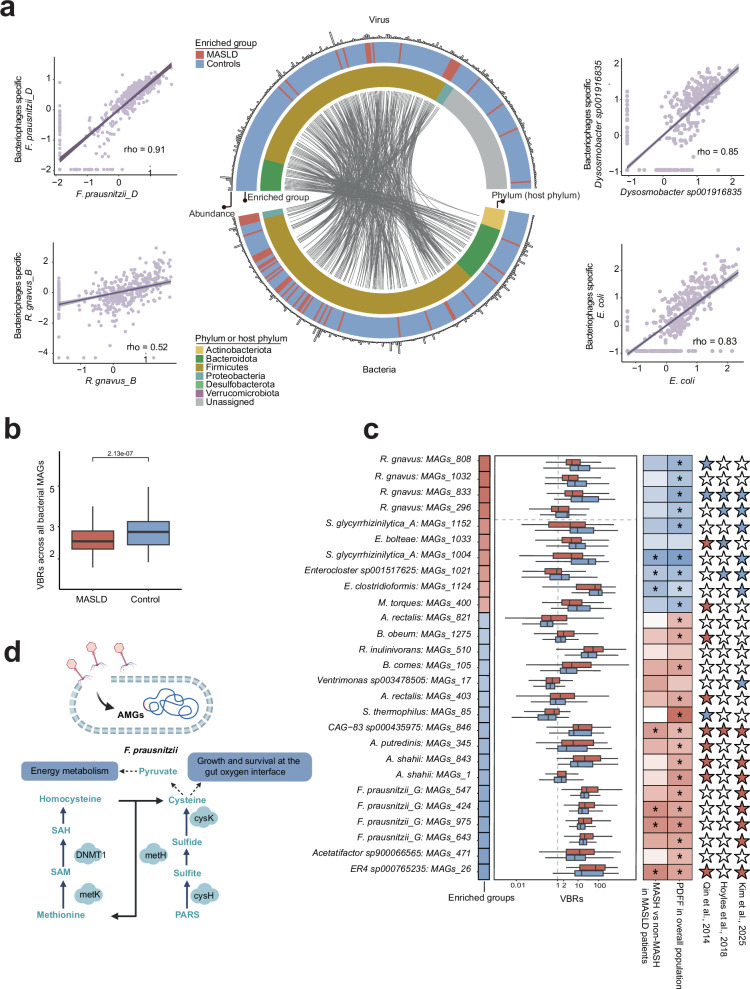
The gut virome dynamics shape microbial ecology. **a** Chord diagram depicting predicted virus-host linkages specific to MASLD-associated viruses and bacterial MAGs. Gray lines denote virus-host connections. The innermost ring is color-coded by bacterial phylum either the MAG’s taxonomy or the predicted host of each virus. The middle ring indicates groups that are significantly enriched (FDR < 0.05). The outer ring displays the prevalence of each microbe. Surrounding the diagram, scatter plots show the two-sided Spearman correlations between four specific bacterial species and their corresponding bacteriophages (all *P* < 0.0001). In each panel, the solid line indicates the fitted linear regression and the shaded band indicates the 95% confidence interval. **b** Distribution of virus-to-bacteria ratios (VBRs) in patients with MASLD (*n* = 210 individuals; red) compared to healthy controls (*n* = 210 individuals; blue) (two-sided Wilcoxon rank-sum test). **c** In total, 27 bacterial MAGs exhibiting opposite directions of association between their VBRs and bacterial abundances in relation to MASLD (210 MASLD patients vs. 210 healthy controls). The VBRs of these MAGs are also associated with disease severity and show consistent alterations across independent validation cohorts. The left heatmap shows differences in bacterial relative abundance between MASLD and control groups (two-sided Wilcoxon rank-sum test; FDR < 0.05; red enriched in MASLD; blue, depleted in MASLD). The middle boxplots display differential VBRs between MASLD patients (red; *n* = 210 individuals) and controls (blue; *n* = 210 individuals) (two-sided Wilcoxon rank-sum test; FDR < 0.05). Box plots show the median, interquartile range and 1.5 × IQR. The right heatmap summarizes associations of bacterial VBRs with MASH status and liver fat content in the MASLD cohort, together with replications in external cohorts. Red and blue indicate positive and negative associations, respectively. In the MASLD cohort, * indicates *P* < 0.05. In the external cohorts, significance was defined as FDR < 0.05 for cirrhosis in Qin et al. and MASLD in Kim et al., and *P* < 0.05 for severe steatosis in Hoyles et al. White stars indicate non-significant association. **d** The schematic illustrates key AMGs involved in methionine and cysteine metabolism (icons created in BioRender. Zhou, X. (2026) https://BioRender.com/2cgs072), which may enhance bacterial energy acquisition and survival, thereby facilitating microbial adaptation within the MASLD-associated gut environment.

To investigate how phage-bacteriome interactions contribute to MASLD-associated dysbiosis, we first characterized 381 robust virus-bacterium linkages based on host prediction at the individual MAG level (Fig. 4a). These MAG-level associations were subsequently summarized at the species level for interpretability. At the species level, we found that 40 (83%) of 48 bacteriophage-bacterium associations were positive (mean Spearman’s rho = 0.48, FDR < 0.05; Supplementary Fig. 2), suggesting widespread co-abundance. Of note, *F. prausnitzii*, a keystone butyrate producers depleted in MASLD, and its phages exhibited strong synchrony, and similar tight phage-host coupling was also observed for *Dysosmobacter sp001916835, E. coli*, and *R. gnavus* (Fig. 4a).

We next assessed phage lytic activity using virus-to-bacteria ratios (VBRs)^29^, which were significantly lower in MASLD than controls (*P* < 0.0001, Fig. 4b), suggesting reduced phage-induced lysis in disease. Among the 161 MASLD-associated bacterial MAGs, 45 showed significant VBR differences between patients and controls, with 27 showing opposing directions of association with MASLD for VBRs and for bacterial abundance (Fig. 4c). For example, *R. gnavus* MAGs were enriched in MASLD and exhibited decreased VBRs, whereas MAGs from *Alistipes. putredinis*, a health-associated species, showed elevated VBRs and was depleted in patients. These patterns were consistently observed across MASLD severity indicators and validated in external datasets, including those involving cirrhosis (Qin et al.)^26^, hepatic steatosis (Hoyles et al.)^27^, and MASLD (Kim et al.)^28^ (Fig. 4c). Prophage activity analysis^30^ further supported this trend (Supplementary Fig. 5a), showing diminished lytic activity in MASLD-associated *R. gnavus* phages and increased activity in phages targeting potentially beneficial taxa like *A. putredinis* (Supplementary Fig. 5b, c).

To assess the functional implications of phage disruption, we profiled auxiliary metabolic genes (AMGs) in viral genomes. Among 264 identified AMGs, 54 were significantly altered in MASLD (*P* < 0.05, Supplementary Fig. 6a), those involved in amino acid, carbohydrate, and vitamin/cofactor metabolism. Notably, genes involved in sulfur-containing amino acid metabolism (*metK*, *metH*, *DNMT1*, *cysH*, and *cysK*) were markedly depleted in MASLD, predominantly carried by phages infecting *F. prausnitzii*, and their abundance correlated with that of *F. prausnitzii* itself (Supplementary Fig. 6a–c), suggesting coordinated regulation of sulfur-containing amino acid metabolism. These AMGs are critical for methionine and cysteine pathways. Given cysteine’s critical roles in energy production^31^, and as an alternative electron acceptor that facilitates the growth and survival of *F. prausnitzii*^32^, the depletion of these AMGs may compromise the ecological fitness of beneficial bacteria in patients with MASLD (Fig. 4d).

Using peak-to-through ratio (PTR) analysis^33^, we assessed bacterial growth rates across 202 species-level MAGs. MASLD patients exhibited elevated growth rates compared to healthy controls (Supplementary Fig. 7a). Among these, 54 MAGs exhibited higher growth rates in MASLD (*P* < 0.05/202, Supplementary Fig. 7b and Supplementary Data 7). *R. gnavus (MAG_1032)* exhibited a significantly higher growth rate in MASLD patients compared with healthy controls (Supplementary Data 7), providing additional evidence for its expansion in disease. These findings collectively suggest disrupted bacteriophage-bacterial interactions and bacterial replication dynamics in MASLD.

### Microbial metabolic shifts link gut dysbiosis to MASLD pathophysiology

**Fig. 5:**
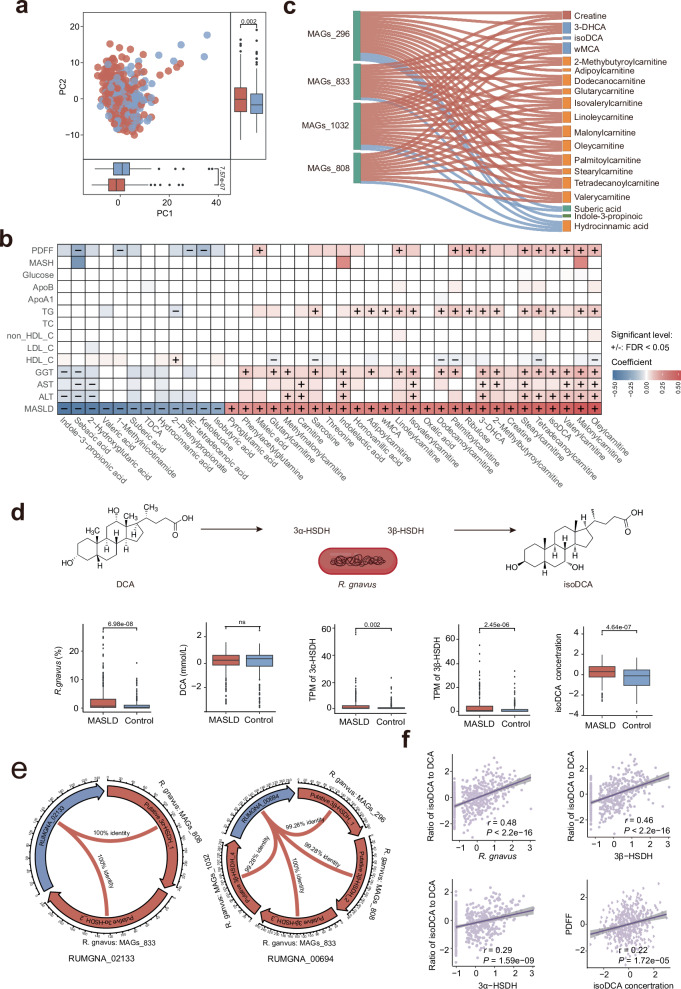
Fecal metabolite isoDCA mediates the association of *R. gnavus* with MASLD. **a** The PCA (principal component analysis) plot shows distinct fecal metabolomic profiles between MASLD (*n* = 207) and control group (*n* = 207). Global differences are assessed using PERMANOVA based on Euclidean distance, and the comparisons of PC1(principal component) and PC2 of fecal metabolome are conducted using two-sided Wilcoxon rank-sum test. Box plots show the median, interquartile range and 1.5 × IQR; outliers are shown as points. **b** Associations between the MASLD-associated metabolites and clinical traits and disease severity indicators. Cells are colored by direction and significance of association (linear regression models adjusted for age, gender and BMI. red: positive with *P* < 0.05, blue: negative with *P* < 0.05, +, −: positive and negative associations respectively with FDR < 0.05). **c** Metabolites significantly associated with *R. gnavus* MAGs (linear regression, FDR < 0.05, |coefficient | > 0.3). **d** Coordinated changes in the abundance of gut bacteria, metabolites and key enzymes involved in the iso-bile acid pathway altered correspondingly. From left to right: changes of *R. gnavus* (210 MASLD vs. 210 controls), DCA (207 MASLD vs. 207 controls), 3α-HSDH (210 MASLD vs. 210 controls), isoDCA (207 MASLD vs. 207 controls) and 3β-HSDH (210 MASLD vs. 210 controls) (two-sided Wilcoxon rank-sum test). Box plots show the median, interquartile range and 1.5 × IQR; outliers are shown as points. Each data point represents one independent participant. Element representing *R. gnavus* is created with BioRender (in BioRender. Zhou, X. (2026) https://BioRender.com/2cgs072). **e** Genomic synteny of four *R. gnavus* MAGs that shared high sequence identity (above 90%) with the reference genes RUMGNA_02133 and RUMGNA_00694. Each ribbon represents the homologous syntenic region between the MAGs belonging to *R.gnavus* and the references. **f** The abundance of *R. gnavus* and its encoded enzymes (3α-HSDH and 3β-HSDH) are highly correlated with the ratio of isoDCA to DCA (data were inverse-normal transformed). Additionally, fecal isoDCA levels are positively correlated with PDFF (two-sided Pearson correlation test, *r* = 0.22, *P* = 1.72 × 10^−5^). In each panel, the solid line indicates the fitted linear regression and the shaded band indicates the 95% confidence interval.

Fecal metabolomic profiling revealed distinct metabolic profiles in MASLD patients (*P* < 0.001, Fig. 5a), and identified 39 differential metabolites (FDR < 0.05), mainly carnitines, amino acids, and bile acids (Fig. 5b). Integrative analysis using Procrustes transformation revealed global correlations between fecal metabolite profiles and gut microbiota across bacteria, viruses, and fungi (all *P* < 0.001), and 28 metabolites were significantly predicted by microbial features, particularly bacterial taxa (Supplementary Fig. 8a, b).

A majority of these differential metabolites were significantly associated with clinical traits and disease severity. Among them, isodeoxycholic acid (isoDCA), a secondary bile acid implicated in liver ballooning^34^ and type 2 diabetes^35^, was significantly elevated in MASLD patients and positively associated with liver fat content (FDR < 0.05, Fig. 5b). Of note, the level of its precursor, deoxycholic acid (DCA), remained unchanged (Fig. 5d), suggesting enhanced enzymatic conversion. This transformation is catalyzed by 3α-hydroxysteroid dehydrogenase (3α-HSDH) and 3β-HSDH, both encoded in *R. gnavus* genomes^36^, whose abundance as well as the levels of 3α/β-HSDH enzymes were enriched in MASLD (Fig. 5d). Genomic analysis further identified these genes in four MAGs from *R. gnavus* (Fig. 5e), whose abundance correlated with isoDCA/DCA ratios (Fig. 5f) and ranked among the top contributors to MASLD-associated metabolites, including isoDCA (Supplementary Fig. 8c and Fig. 5c). These findings support a potential role of *R. gnavus*-derived HSDHs in bile acid dysregulation and MASLD pathogenesis.

### Microbial signatures improve MASLD classification

**Fig. 6:**
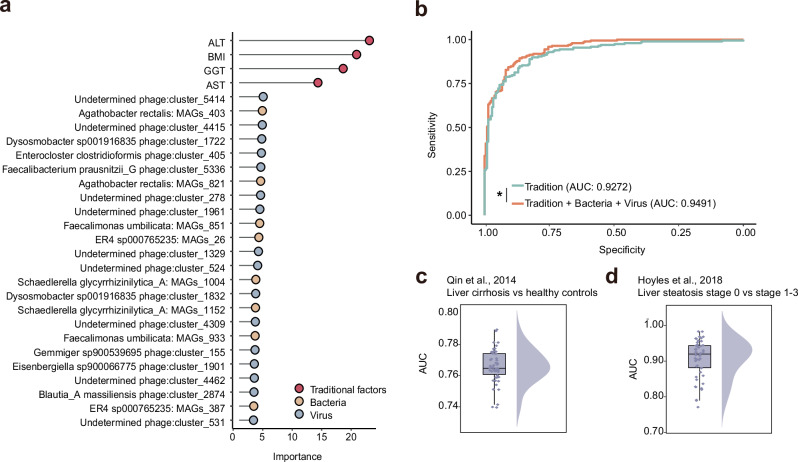
Microbial signatures demonstrated powerful discriminatory power for distinguishing MASLD patients from healthy individuals. **a** Importance ranking of 28 selected features in the MASLD classification model. Features are ordered by their contribution to model performance. **b** Receiver operating characteristic (ROC) curves comparing the performance of classification models based on traditional clinical variables alone (BMI, ALT, AST, and GGT; area under the curve (AUC) = 0.93) and a combined model that additionally incorporates bacterial and viral signatures (AUC = 0.95). The inclusion of microbial features significantly improves model performance (two-sided Delong test *P* = 0.02). **c**, **d** ROC curves showing the discriminatory performance of the 24 selected microbial biomarkers in two external validation datasets for **c** liver cirrhosis status (98 cirrhosis patients and 83 controls) and **d** severity stages of liver steatosis (10 individuals with stage 0–1 steatosis and 46 with advanced steatosis). The central band of the box plot represents the median AUC of 50 iterations, the box limits indicate the first and third quartiles, and the whiskers extend to 1.5× interquartile range.

To develop a robust non-invasive diagnostic classifier for MASLD, we evaluated multiple machine algorithms and selected gradient boosting based on its superior (with an area under the curve (AUC) of 0.94, Supplementary Fig. 9). Using this framework, we constructed a classifier of 28 informative features, including BMI, liver enzymes, and specific gut bacterial and viral taxa, identified by the Boruta feature selection procedure (Fig. 6a). The classification model achieved an AUC of 0.95, significantly outperforming a clinical-only model (Delong test *P* = 0.02, Fig. 6b).

Using microbial features only, we evaluated the reproducibility of the biomarker panel in independent external datasets. The microbial features achieved a mean AUC of 0.77 in the cirrhosis cohort from Qin et al. (Fig. 6c) and a mean AUC of 0.91 in the steatosis severity cohort from Hoyles et al. (Fig. 6d). In the dataset from Kim et al., these microbial features achieved moderate discriminatory performance between MASLD patients and controls (mean AUC = 0.62; Supplementary Fig. 10), comparable to that reported in the original model by Kim et al. (AUC = 0.65)^28^. By contrast, when the model trained in our discovery cohort was directly applied to external datasets without re-training, its performance was more modest, with AUC values ranging from 0.51 to 0.58, indicating that although the selected microbial markers are reproducible across cohorts, the original discovery-trained model has limited generalizability. Overall, these results highlight the potential utility of gut microbiome signatures as non-invasive biomarkers for MASLD detection and progression monitoring.

### Subgroup analysis of MASLD patients with cardiometabolic risk factors

To assess robustness to updated diagnostic criteria, we repeated key analyses in the subset of MASLD patients with ≥1 cardiometabolic risk factor^37^ (MASLD_CAR, *n* = 196). The overall patterns of microbiome, virome, and metabolite associations remained highly consistent with the full MASLD cohort, including the key differential taxa, VBR patterns, isoDCA difference, and ROC curve (Supplementary Fig. 11).

## Discussion

In this large, well-matched case-control study, we provide a comprehensive characterization of multi-kingdom gut dysbiosis in MASLD within an East Asian population, extending and complementing prior findings by Kim et al. in a Caucasian cohort^28^. Moreover, our study reveals additional insights into phage-bacteria ecological interactions linked to bile acid metabolism, uncovering a previously unrecognized phage-bacterium-metabolite axis relevant to MASLD progression. Our findings reveal coordinated shifts in bacteriophage–bacteria dynamics, accompanied by changes in microbial growth, metabolic activity, and disruptions in host-microbe metabolic signaling. Furthermore, we developed and externally validated a microbiome-based classifier that robustly distinguishes MASLD patients from controls, demonstrating strong generalizability across independent populations.

At the compositional level, we observed a consistent reduction in microbial α-diversity and extensive compositional remodeling across bacterial, viral, and fungal communities in MASLD, potentially increasing susceptibility to pathobiont overgrowth^38^. Taxonomically, a key finding was the coordination between bacteriophages and their bacterial hosts. In particular, phages targeting *E. coli* and *R. gnavus*, both enriched in MASLD, were also elevated, while phages infecting beneficial taxa such as *F. prausnitzii* and *F. saccharivorans* were depleted. Of note, MAGs from *F. prausnitzii* and *R. gnavus*, along with their bacteriophages, displayed consistent associations with disease severity, suggesting synchronized microbial shifts along the MASLD-cirrhosis continuum. These virome-bacteriome imbalances may destabilize the gut ecosystem and represent an ecological mechanism linking virome perturbations to host metabolic dysfunction, a phage-centric restructuring that is particularly compelling in light of the growing recognition of bacteriophages as key regulators of bacterial phenotypes, microbial community structure, and host physiology^39^. Complementing these findings, mycobiome analyses revealed MASLD-specific taxonomic shifts, including depletion of *Penicillium* and enrichment of *Alternaria*, a genus linked to lipid metabolism^23^. This underscores the metabolic relevance of fungi in MASLD pathogenesis, and highlight the need to study cross-kingdom interactions. Beyond taxonomic alterations, we further observed profound changes in microbial network topology in MASLD. Despite reduced α-diversity, both intra- and inter-kingdom networks were much denser in the MASLD patients. This pattern aligns with reports in chronic inflammatory diseases, such as chronic obstructive pulmonary disease^40^ and colorectal cancer^41^, suggesting a convergent ecological response to host inflammation and dysbiosis. Collectively, these findings emphasize that multi-kingdom microbial interactions are critical to understanding gut-liver axis dysregulation in MASLD.

Of note, *R. gnavus*, emerged as a central microbial hub enriched in MASLD patients, accompanied by reduced lytic activity of *R. gnavus-*targeting phages in both MASLD and cirrhosis, suggesting diminished viral control. This overgrowth is biologically plausible, as *R. gnavus* has been shown to upregulate hepatic lipid synthesis genes^42^ and impair insulin sensitivity^43^. Meanwhile, key commensal bacteria such as *F. prausnitzii*, *A.shahii*, and *A. putredinis* exhibited increased phage lytic activity, potentially contributing to their depletion in MASLD. Compounding this imbalance, *F. prausnitzii* may experience additional fitness compromise in MASLD from the loss of phage-encoded AMGs, particularly those regulating sulfur amino acid metabolism, a process critical for energy acquisition and redox balance in anaerobic bacteria^32^. In contrast, *R. gnavus* phages displayed lower lytic activity, potentially facilitating its overgrowth in MASLD. Together with reduced VBRs and elevated bacterial growth rates in MASLD, these phage-mediated functional constraints appear to drive the selective expansion of pathobionts and loss of beneficial commensals, thereby promoting host metabolic dysregulation.

We previously demonstrated that gut dysbiosis promotes nonalcoholic fatty liver disease via increasing circulating levels of secondary bile acids^3^. Here, we extend those findings by demonstrating that MASLD patients exhibited increased fecal isoDCA, a secondary bile acid derived from DCA and implicated in hepatocellular ballooning^34^, while the level of its precursor, DCA, remain unchanged. This rise in isoDCA corresponds with marked enrichment in MASLD of *R. gnavus*, a species known to convert DCA to isoDCA^44^, alongside reduced lytic activity of *R. gnavus*-targeting phages in both MASLD and cirrhosis. Collectively, these observations support a mechanistic phage-bacteria-metabolite axis in MASLD, characterized by impaired phage regulation, expansion of bile acid-modifying pathobionts, depletion of protective commensals, and disruption of bile acid homeostasis.

Leveraging our multi-kingdom microbial insights, we developed a dedicated microbiome-based classifier for MASLD. Our model identified a set of bacterial and viral features that distinguished MASLD patients from healthy controls with a high accuracy (AUC = 0.95) and outperformed a traditional classifier (AUC = 0.93). These microbial features were not only associated with disease presence but also correlated with imaging-derived indicators of liver fat accumulation and fibrosis severity, reinforcing their clinical relevance. Notably, the classifier maintained robust classification performance in two external cohorts, including patients with cirrhosis and biopsy-confirmed steatosis, demonstrating strong generalizability across disease stages. When applied to the dataset from Kim et al., the classifier achieved moderate discrimination between MASLD patients from controls, comparable to the performance of the original model reported by the authors, likely reflecting the self-reported nature of case definitions in that study^28^. Together, these results support the use of gut microbial signatures as scalable, non-invasive tools for MASLD detection and staging. Future studies leveraging marker genes from the selected microbial MAGs and prophages, or targeted qPCR panels, may enable rapid and standardized diagnostic classification.

The study has several limitations. First, the primary study population was drawn from a Chinese population at a single time point, and although we validated key findings in three external cohorts, future longitudinal studies in larger, ethnically and geographically diverse populations are needed to confirm the generalizability of the proposed phage-bacteria-metabolite axis. Second, diet, medications (e.g., proton pump inhibitors, metformin), and other lifestyle factors can profoundly shape the microbiome and metabolome. Although medication use was minimal and analyses were adjusted for age, gender, and BMI, residual confounding, particularly by diet, a dominant determinant of gut microbiome composition and a key driver of MASLD pathogenesis, cannot be excluded, and future studies incorporating standardized dietary assessments and longitudinal designs will be required to disentangle diet-driven effects from disease-associated microbiome alterations and to establish causality. Third, while we integrated bacteriome, virome, mycobiome, and metabolome data to support a mechanistic framework linking disrupted phage regulation with microbial imbalance and metabolic perturbations in MASLD, direct causal evidence remained limited. Targeted experimental models will be essential to delineate the molecular pathways by which phage-host interactions drive disease onset and progression. While our metabolomic analyses focused on fecal samples to capture gut microbiome-derived metabolic activity, integration of blood metabolomics in future studies will be essential to link local microbial metabolism with systemic host metabolic responses and MASLD progression.

In summary, our multi-kingdom, multi-omics analysis reveals a mechanistic phage-bacteria-metabolite axis underlying MASLD pathogenesis. This axis is characterized by disrupted phage-mediated regulation, overgrowth of bile acid-modifying pathobionts, loss of beneficial commensals, and altered microbial metabolism, particularly in bile acid pathways. These insights deepen our understanding of the ecological and functional foundations of MASLD and provide a mechanistic basis for developing phage-based therapeutic strategies, suggesting that restoring phage-bacteria dynamics may offer a potential approach for MASLD management.

## Methods

### Study design and imaging-based evaluation

Between 2021 and 2022, 210 clinically diagnosed MASLD patients were recruited from Zhongshan Hospital in Shanghai, China. An additional 210 healthy individuals, matched for gender and age (±5 years), were selected from a healthy cohort recruited at the same hospital during the same period. Abdominal ultrasonography was performed on all participants to confirm the presence of MASLD. Participants were excluded if they had significant alcohol consumption (defined as ≥20 g per day for men and ≥10 g per day for women); other liver diseases; kidney disorders; cancer or other serious illnesses; had undergone major surgery within the previous 8 weeks; or had used antibiotics within the prior 4 weeks. Data on comorbidities and medication use among MASLD patients were collected from medical records and self-reports. No statistical method was used to predetermine sample size.

Magnetic resonance imaging proton density fat fraction (MRI-PDFF) was measured on 366 participants using a Siemens 1.5 T MAGNETOM Aera. MR data were analysed using LiverMultiScan™ Discover software. PDFF maps were constructed using the second, fourth, and sixth of the ten MR echoes, using a three-point DIXON technique. Liver elastography was conducted in 172 MASLD patients using the FibroScan 502 TOUCH device (Echosens, France) to assess liver stiffness. Examinations were performed in the fasting state by an experienced gastroenterologist blinded to clinical information, with patients positioned in the standard supine posture. For individuals with a BMI (body mass index) >30 kg/m², the XL probe was used; otherwise, the M probe was applied. Both the controlled attenuation parameter (CAP) and liver stiffness measurement (LSM) were recorded to evaluate the severity of MASLD.

This study was approved by the Ethics Committee of Zhongshan Hospital (No: B2020-085) and the Research Ethics Committee of the School of Life Sciences at Fudan University (No: FE24199I). Written informed consent was obtained from all participants.

### Anthropometric and serum biochemical examinations

Body weight and height were measured with participants barefoot, and BMI was calculated as body weight (kg) divided by the square of height (m^2^). Fasting blood biochemical parameters, including alanine aminotransferase, aspartate aminotransferase, gamma-glutamyl transferase, total cholesterol, triglycerides, HDL-C, LDL-C, total bilirubin, and direct bilirubin, were measured using an automated bioanalyzer (HITACHI 7600, Japan).

### Fecal samples processing, gut microbial and metabolite profiling

Fecal samples were collected from all participants at enrollment, immediately stored at 4 °C, and then transferred to –80 °C storage within 2 h. Fecal DNA was extracted using the DNeasy 96 PowerSoil Pro QIAcube HT Kit (Qiagen, Germany) on the QIAcude automated platform according to the manufacturer’s instructions. Subsequent DNA libraries were prepared with the DNA Library Prep Kit (APExBIO, USA) on the Biomek i5 automated workstation^45^. Shotgun metagenomic sequencing was conducted on the Illumina Novaseq 6000 (paired-end 250 bp) sequencing platform, yielding approximately 10 Gb of reads per sample.

The gut mycobiome was profiled using internal transcribed spacer 2 (ITS2) sequencing of fecal samples from participants. The ITS2 hypervariable regions were amplified with primers ITS3F (GCATCGATGAAGAACGCAGC) and ITS4R (TCCTCCGCTTATTGATATGC) on an ABI GeneAmp 9700 PCR thermocycler (ABI, USA). The PCR amplification was conducted as the following programs: initial denaturation at 95 °C for 3 min, followed by 27 cycles of denaturing at 95 °C for 30 s, annealing at 55 °C for 30 s, and elongation at 72 °C for 45 s, with a final extension at 72 °C for 10 min. The PCR products were then extracted from a 2% agarose gel and purified using the AxyPrep DNA Gel Extraction Kit (Axygen Biosciences, USA), and quantified by QuantusTM Fluorometer (Promega, USA) following the manufacturer’s instructions. Purified amplicons were pooled in equimolar amounts and sequenced on MiSeq PE300 platform (Illumina, USA). Raw reads were demultiplexed, subjected to quality control using fastp^46^, and processed with QIIME2^47^. Remained high-quality sequences were denoised and clustered into amplicon sequence variants (ASVs) via DADA2^48^. Taxonomic classification of fungal ASVs was implemented using Naïve Bayes classifier against the UNITE database (v9.0)^49^. Rarefaction was conducted on samples reaching a sequencing depth of 10,000 reads.

Targeted fecal metabolomics was conducted using the Q300 Metabolite Array Kit (METABO-Profile, China)^50^. Briefly, metabolite profiling was conducted by an ultra-performance liquid chromatography coupled to tandem mass spectrometry (UPLC-MS/MS) system (Waters Corp., USA). Of the 214 fecal metabolites were detected, three were excluded due to the high variability (coefficient of variation >30%) in quality control samples. For the remaining metabolites, values below the detection limit were imputed as half of the minimal detected value. Metabolites’ concentrations were then Log_10_ transformed and standardized (Z-score transformation with mean = 0 and variance = 1) prior to statistical analysis. These metabolites were annotated as microbially associated or host-specific based on the classification by Hua et al.’s^51^ for the Q300 panel (Supplementary Data 8).

### Establishment of bacterial MAGs and vOTUs

Shotgun metagenomic reads were quality-filtered for the removal of low-quality and host-derived reads using KneadData^52^, retaining an average of 65.53 million (standard deviation: 9.63) high-quality reads per sample. De novo assembly was conducted on each sample individually using MegaHIT (v1.2.9, --minlen 500)^53^. All sample-specific contigs were aggregated into a unified contig catalogue.

Reads were then mapped to contig catalogue using BWA-mem (v0.7.17)^54^ with default parameters. Per-sample contig abundance profiles were calculated using jgi_summarize_bam_contig_depths script from MetaBAT2^55^. VAMB (v3.1)^56^ was employed to cluster the contigs into putative biological entities (e.g., bacteria and viruses) using the jgi-depth matrix and tetranucleotide frequencies derived from input sequences as inputs. The quality of bacterial bins was determined using the lineage-wf workflow from CheckM (v1.3.3)^57^, and only high-quality bins (completeness ≥ 95%, contamination ≤5%, and strain heterogeneity ≤5%) were retained for further analysis (*n* = 2231). All these bins were then dereplicated at 99% average nucleotide identity (ANI) level using dRep (v3.4.0)^58^, yielding 1344 metagenome-assembled genomes (MAGs). Taxonomic annotation of each MAG was assigned with GTDB-TK (v2.1.0) based on the GTDBTK database (release 207)^15^. MAGs that could not be assigned to any known species were further determined whether represented previously undescribed species by mapping them against the species-level genome bins (SGBs) database (SGB.Oct19)^59^ using PhyloPhlAn 3^16^. Notably, three MAGs failed to cluster with any known SGBs at 5% genetic distance threshold, suggesting potentially new bacterial taxa.

Viral genomes were identified from VAMB bins using PHAMB^17^, and completeness was estimated with CheckV^60^, considering only AAI-based estimates. Viral bins with genome copy number ≥1.25 or completeness >120% were excluded. Remaining bins meeting medium (≥ 50% completeness), high (≥ 90% completeness), and complete (closed genomes) quality standards were clustered into 5440 viral operational taxonomic units (vOTUs)^60^.

Furthermore, we also parsed vOTUs using the VIBRANT^61^ (v1.2.1, with default parameters), which showed a 93.93% viral prediction agreement (5110/5440) with the PHAMB-derived vOTUs.

Viral proteins were predicted with Prodigal^62^, and annotated using the Demovir (https://github.com/feargalr/Demovir) with TrEMBL database. The taxonomy of a vOTU was assigned if more than two proteins within this viral genome reached a majority vote (≥ 50%) and were annotated with the same taxonomy.

To test whether the vOTUs were previously described, we clustered vOTUs with viral genomes from the Unified Human Gut Virome Catalog (UHGV; https://github.com/snayfach/UHGV) using the methods we clustered the viral genomes into vOTUs. vOTUs clustered with viral genomes from UHGV were annotated as ‘known viruses,’ while others were annotated as ‘previously undescribed.’

### Estimation of bacteria MAGs and vOTU abundance

The abundance of MAGs and vOTUs was estimated using CoverM^63^. Clean reads of each sample were mapped to genomes of MAGs and vOTUs using the CoverM contig module to estimate the abundance and reads per kilobase per million mapped reads of the MAGs and viral clusters separately.

### Prediction of viral hosts

Hosts for the viral genomes were determined by the combination of CRISPR spacers and sequence similarity. First, CRISPR spacers were identified from the MAGs using CrisprCasTyper (v1.2.3)^64^ with the ‘--prodigal meta’ mode. Spacer sequences were then blasted against viral genomes, retaining matches with ≥ 95% sequence identity over ≥95% of the spacer length and a maximum of two mismatches. In parallel, viral bins were aligned to MAGs using FastANI^65^ with ANI ≥ 90% based on at least one 5000 bp fragment. Based on these predictions, vOTUs were annotated with the corresponding bacterial hosts species, while those without identifiable hosts were labeled as ‘undetermined’ (41.53%).

To explore bacterial host–virus abundance relationships, the relative abundance of MAGs within the same species were aggregated, as were the abundances of vOTUs predicted to infect them. Spearman correlations analysis as performed to assess associations between these paired summed values.

### Estimation of prophage activity

To assess the influence of gut bacteriophages on their bacterial hosts, prophage activity was estimated by two complementary methods. First, the virus-to-bacteria ratio (VBR) was calculated for each bacterial MAGs by dividing the total abundance of bacteriophages by the abundance of their corresponding host MAG. Second, PropagAtE (v1.1.0)^30^ was applied in prophage activity analysis by comparing the read coverage of the integrated prophage regions to that of their adjacent host genomic regions.

### Quantification of the viral metabolic potentials

Prodigal^62^ (v2.6.3) was utilized to predict genes of each contig obtained with settings:-p meta. A non-redundant gene catalogue was constructed by clustering predicted genes with CD-HIT (-c 0.95 -aS 0.9)^66^.

Auxiliary metabolic genes (AMGs) were firstly mined from the gene catalogue with VIBRANT (v1.2.1)^61^. Quality-filtered reads were mapped to the gene catalogue with BWA-mem^54^ (v 0.7.17-r1188) with default parameters. Gene abundance was quantified as the transcripts per kilobase per million (TPM), providing a measure of the relative abundance of phage-encoded AMGs in our study population.

### Estimation of bacteria growth rates

Bacterial growth rates were quantified by estimating peak-to-trough ratios (PTRs) using CoPTR (v1.1.6)^33^ for MAGs in each individual sample. As CoPTR provides species-level PTRs estimates, MAGs were initially re-clustered at 95% ANI, yielding 343 bacterial genomes. Clean reads were subsequently mapped against these bacterial genomes, and the combined coverage profiles were used to calculate PTRs. To enhance the robustness of our estimates, only MAGs detected in over 50 individuals (*n* = 202) were included for PTR analysis.

### Constructions of cohort-specific microbial networks

Microbial interaction networks were constructed separately for the MASLD and control groups. Partial correlation was computed among microbial features, adjusted for age, gender and BMI. *P* values were corrected for multiple comparisons using Benjamini–Hochberg procedure. Only correlations with an absolute rho value larger than 0.4 were retained for networks construction. The topological characteristics of each node were subsequently calculated using the R package *igraph*^67^.

### Replication datasets

To investigate whether microbial features distinguishing MASLD patients from healthy controls also validated in external MASLD dataset and associated with the disease severity, we extended our analysis to external datasets. Replication dataset 1 comprised 181 individuals (98 patients with liver cirrhosis and 83 healthy controls), as reported by Qin et al.^26^. Replication dataset 2 was comprising of 56 biopsy-proven MASLD patients, including 10 with stage 0–1 steatosis and the remainder with more advanced steatosis^27^. Replication Dataset 3, including 211 MASLD patients and 502 controls from a cohort of female Caucasian participants^28^.

Metagenomic sequencing data from fecal samples were retrieved from the European Nucleotide Archive at the European Bioinformatics Institute (accession numbers: PRJEB6337, PRJEB14215, and PRJNA1246224, respectively). We profiled the abundances of bacteria and virus using coverM, based on the same reference database constructed for our study population. Microbiome features associated with fibrosis and MASLD in replication datasets 1 and 3 were identified using linear regression models adjusted for age, gender (dataset 1 only), and BMI. For dataset 2, no adjustments were applied for this cohort because covariate information is unavailable. Group differences were compared by applying Wilcoxon rank-sum test.

### Identification and quantification of bacterial enzymes in the iso-bile acid pathway

Two bacterial enzymes are involved in conversion of DCA (deoxycholic acid) to isoDCA (isodeoxycholic acid): 3α-hydroxysteroid dehydrogenases (3α-HSDH), which transforms DCA into 3-oxoDCA (3-oxo deoxycholic acid), and 3β-HSDH, which subsequently converts 3-oxoDCA to isoDCA. In this study, genes encoding these two enzymes were identified across 1,344 bacterial MAGs via BLASTx search against the amino acid sequence of RUMGNA_02133 and RUMGNA_00694 (HSDHs present in the *Ruminococcus gnavus*)^44^.

To quantify the abundance of 3α-HSDH and 3β-HSDH, the gene catalogue was queried using BLASTp against the same reference sequences. Genes with ≥ 80% identity and *e*-value < 1e-5 were considered homologous, and their abundances were summed to estimate the abundance of 3α-HSDH and 3β-HSDH.

### Construction of microbiome-based classification models

To identify an appropriate modeling framework for MASLD classification, we first performed a model evaluation step by benchmarking multiple widely used supervised machine learning algorithms, including logistic regression with L1 and L2 regularization, decision tree, random forest, gradient boosting, k-nearest neighbors, and support vector classification. During this initial evaluation, all available microbial features were included and default model parameters were used to ensure a fair comparison across algorithms. Model performance was assessed using cross-validation AUC. Based on this evaluation, gradient boosting was selected as the primary modeling approach for subsequent analyses. Following model selection, informative microbial features and clinical traits were identified using the Boruta feature selection algorithm. Hyperparameters of the gradient boosting model, including the number of estimator trees, the maximum depth of the trees, the numbers of features per tree, and the maximum samples were then optimized via Bayesian optimization with cross-validation AUC as the objective function to construct the final best-performing classifier. All procedures were performed using xMarkerFinder^68^.

For external validations, microbial markers identified in our primary study were used to retrain and optimize the models within each validation dataset. This process was repeated 50 times to ensure model robustness. In Replication Dataset 2 from Hoyles et al.^27^, where the number of individuals with stage 0 liver steatosis was limited (*n* = 10), random oversampling was applied to increase the sample size to 20, enabling balanced model training.

### Statistical analysis

Associations between gut microbial features, fecal metabolites, and MASLD status were evaluated using linear regression models adjusted for age, gender, and body mass index (BMI). Prior to statistical analysis, only microbial taxa with a minimum relative abundance of 0.01% in more than 10% of samples were included. All microbiome and metabolite data were log-transformed, and Z-score standardized to facilitate interpretability and comparability of regression coefficients. Comparisons between two groups were performed using two-sided Wilcoxon rank-sum tests unless otherwise specified, and overall community differences were evaluated using PERMANOVA where appropriate. Multiple-comparison correction was performed using the Benjamini–Hochberg method to control the false discovery rate (FDR). Statistical details for individual analyses are provided in the corresponding figure legends.

### Reporting summary

Further information on research design is available in the Nature Portfolio Reporting Summary linked to this article.

## Supplementary information

Supplementary_information

Description of Additional Supplementary Files

Supplementary dataset 1–8

Reporting Summary

Transparent Peer Review file

## Source data

Source Data

## Data Availability

Raw metagenomic sequencing data, accompanied by metadata including age, gender, and disease status, have been deposited in the Genome Sequence Archive (GSA) under accession code PRJCA060134. This BioProject also includes the assembled bacterial MAGs generated in this study. Metagenomic sequencing data and MAGs are also available at the National Omics Data Encyclopedia under accession number OEP00005883. Metagenomic sequencing data for external validation datasets were retrieved from the European Nucleotide Archive at the European Bioinformatics Institute (accession numbers: PRJEB6337, PRJEB14215, and PRJNA1246224, respectively). Raw metabolomic data have been deposited in MetaboLights under accession code MTBLS14073. Source data are provided with this paper.

## Code availability

Codes used for the main figures have been deposited in Zenodo (10.5281/zenodo.19097339).

## References

[1] Paik, J. M. et al. Changes in the global burden of chronic liver diseases from 2012 to 2017: the growing impact of NAFLD. *Hepatology* **72**, 1605–1616 (2020). 32043613 10.1002/hep.31173

[2] Younossi, Z. M. et al. The global epidemiology of nonalcoholic fatty liver disease (NAFLD) and nonalcoholic steatohepatitis (NASH): a systematic review. *Hepatology* **77**, 1335–1347 (2023). 36626630 10.1097/HEP.0000000000000004 PMC10026948

[3] Jiao, N. et al. Suppressed hepatic bile acid signalling despite elevated production of primary and secondary bile acids in NAFLD. *Gut* **67**, 1881–1891 (2018). 28774887 10.1136/gutjnl-2017-314307

[4] Liu, Y. et al. Early prediction of incident liver disease using conventional risk factors and gut-microbiome-augmented gradient boosting. *Cell Metab.* **34**, 719–730.e4 (2022). 35354069 10.1016/j.cmet.2022.03.002 PMC9097589

[5] Loomba, R. et al. Gut microbiome-based metagenomic signature for non-invasive detection of advanced fibrosis in human nonalcoholic fatty liver disease. *Cell Metab.* **25**, 1054–1062.e5 (2017). 28467925 10.1016/j.cmet.2017.04.001 PMC5502730

[6] Zhu, L. et al. Characterization of gut microbiomes in nonalcoholic steatohepatitis (NASH) patients: a connection between endogenous alcohol and NASH. *Hepatology* **57**, 601–609 (2013). 23055155 10.1002/hep.26093

[7] Zhang, X. et al. Dietary cholesterol drives fatty liver-associated liver cancer by modulating gut microbiota and metabolites. *Gut* **70**, 761–774 (2021). 32694178 10.1136/gutjnl-2019-319664 PMC7948195

[8] Wei, W. et al. Parabacteroides distasonis uses dietary inulin to suppress NASH via its metabolite pentadecanoic acid. *Nat. Microbiol.* **8**, 1534–1548 (2023). 37386075 10.1038/s41564-023-01418-7 PMC10390331

[9] Demir, M. et al. The fecal mycobiome in non-alcoholic fatty liver disease. *J. Hepatol.* **76**, 788–799 (2022). 34896404 10.1016/j.jhep.2021.11.029 PMC8981795

[10] Lang, S. et al. Intestinal virome signature associated with severity of nonalcoholic fatty liver disease. *Gastroenterology* **159**, 1839–1852 (2020). 32652145 10.1053/j.gastro.2020.07.005 PMC8404510

[11] Hsu, B. B. et al. Dynamic modulation of the gut microbiota and metabolome by bacteriophages in a mouse model. *Cell Host Microbe* **25**, 803–814.e5 (2019). 31175044 10.1016/j.chom.2019.05.001 PMC6579560

[12] Duan, Y. et al. Bacteriophage targeting of gut bacterium attenuates alcoholic liver disease. *Nature* **575**, 505–511 (2019). 31723265 10.1038/s41586-019-1742-x PMC6872939

[13] Rasmussen, T. S. et al. Faecal virome transplantation decreases symptoms of type 2 diabetes and obesity in a murine model. *Gut* **69**, 2122–2130 (2020). 32165408 10.1136/gutjnl-2019-320005

[14] Jiao, N., Zhu, L. & Zhu, R. The search for authentic microbiome-disease relationships. *Nat. Med.* **30**, 1243–1244 (2024). 38689061 10.1038/s41591-024-02920-z

[15] Chaumeil, P. A. et al. GTDB-Tk v2: memory friendly classification with the genome taxonomy database. *Bioinformatics* **38**, 5315–5316 (2022). 36218463 10.1093/bioinformatics/btac672 PMC9710552

[16] Asnicar, F. et al. Precise phylogenetic analysis of microbial isolates and genomes from metagenomes using PhyloPhlAn 3.0. *Nat. Commun.* **11**, 2500 (2020). 32427907 10.1038/s41467-020-16366-7 PMC7237447

[17] Johansen, J. et al. Genome binning of viral entities from bulk metagenomics data. *Nat. Commun.* **13**, 965 (2022). 35181661 10.1038/s41467-022-28581-5 PMC8857322

[18] Shah, S. A. et al. Expanding known viral diversity in the healthy infant gut. *Nat. Microbiol.* **8**, 986–998 (2023). 37037943 10.1038/s41564-023-01345-7 PMC10159846

[19] Shkoporov, A. N. et al. The human gut virome is highly diverse, stable, and individual specific. *Cell Host Microbe* **26**, 527–541.e5 (2019). 31600503 10.1016/j.chom.2019.09.009

[20] Quevrain, E. et al. Identification of an anti-inflammatory protein from Faecalibacterium prausnitzii, a commensal bacterium deficient in Crohn’s disease. *Gut* **65**, 415–425 (2016). 26045134 10.1136/gutjnl-2014-307649 PMC5136800

[21] Shin, J. H. et al. Faecalibacterium prausnitzii prevents hepatic damage in a mouse model of NASH induced by a high-fructose high-fat diet. *Front. Microbiol.* **14**, 1123547 (2023). 37007480 10.3389/fmicb.2023.1123547 PMC10060964

[22] Munukka, E. et al. *Faecalibacterium prausnitzii* treatment improves hepatic health and reduces adipose tissue inflammation in high-fat fed mice. *ISME J.* **11**, 1667–1679 (2017). 28375212 10.1038/ismej.2017.24 PMC5520144

[23] Shuai, M. et al. Mapping the human gut mycobiome in middle-aged and elderly adults: multiomics insights and implications for host metabolic health. *Gut* **71**, 1812–1820 (2022). 35017200 10.1136/gutjnl-2021-326298 PMC9380515

[24] Marti-Aguado, D. et al. Low-to-moderate alcohol consumption is associated with increased fibrosis in individuals with metabolic dysfunction-associated steatotic liver disease. *J. Hepatol.* **81**, 930–940 (2024). 38971533 10.1016/j.jhep.2024.06.036

[25] Newsome, P. N. et al. FibroScan-AST (FAST) score for the non-invasive identification of patients with non-alcoholic steatohepatitis with significant activity and fibrosis: a prospective derivation and global validation study. *Lancet Gastroenterol. Hepatol.* **5**, 362–373 (2020). 32027858 10.1016/S2468-1253(19)30383-8 PMC7066580

[26] Qin, N. et al. Alterations of the human gut microbiome in liver cirrhosis. *Nature* **513**, 59–64 (2014). 25079328 10.1038/nature13568

[27] Hoyles, L. et al. Molecular phenomics and metagenomics of hepatic steatosis in non-diabetic obese women. *Nat. Med.* **24**, 1070–1080 (2018). 29942096 10.1038/s41591-018-0061-3 PMC6140997

[28] Kim, H. et al. Multi-omic analysis reveals transkingdom gut dysbiosis in metabolic dysfunction-associated steatotic liver disease. *Nat. Metab.* **7**, 1476–1492 (2025). 40604156 10.1038/s42255-025-01318-6 PMC12320953

[29] Liang, G. et al. The stepwise assembly of the neonatal virome is modulated by breastfeeding. *Nature* **581**, 470–474 (2020). 32461640 10.1038/s41586-020-2192-1 PMC7263352

[30] Kieft, K. & Anantharaman, K. Deciphering active prophages from metagenomes. *mSystems* **7**, e0008422 (2022). 35323045 10.1128/msystems.00084-22 PMC9040807

[31] Tchong, S. I., Xu, H. & White, R. H. L-cysteine desulfidase: an [4Fe-4S] enzyme isolated from *Methanocaldococcus jannaschii* that catalyzes the breakdown of L-cysteine into pyruvate, ammonia, and sulfide. *Biochemistry* **44**, 1659–1670 (2005). 15683250 10.1021/bi0484769

[32] Khan, M. T. et al. The gut anaerobe *Faecalibacterium prausnitzii* uses an extracellular electron shuttle to grow at oxic-anoxic interphases. *ISME J.* **6**, 1578–1585 (2012). 22357539 10.1038/ismej.2012.5 PMC3400418

[33] Joseph, T. A. et al. Accurate and robust inference of microbial growth dynamics from metagenomic sequencing reveals personalized growth rates. *Genome Res.* **32**, 558–568 (2022). 34987055 10.1101/gr.275533.121 PMC8896461

[34] Nimer, N. et al. Bile acids profile, histopathological indices and genetic variants for non-alcoholic fatty liver disease progression. *Metabolism* **116**, 154457 (2021). 33275980 10.1016/j.metabol.2020.154457 PMC7856026

[35] Wahlstrom, A. et al. Production of deoxycholic acid by low-abundant microbial species is associated with impaired glucose metabolism. *Nat. Commun.* **15**, 4276 (2024). 38769296 10.1038/s41467-024-48543-3 PMC11106306

[36] Doden, H. L. et al. Completion of the gut microbial epi-bile acid pathway. *Gut Microbes* **13**, 1–20 (2021). 33938389 10.1080/19490976.2021.1907271 PMC8096331

[37] Rinella, M. E. et al. A multisociety Delphi consensus statement on new fatty liver disease nomenclature. *Ann. Hepatol.* **29**, 101133 (2024). 37364816 10.1016/j.aohep.2023.101133

[38] Spragge, F. et al. Microbiome diversity protects against pathogens by nutrient blocking. *Science* **382**, eadj3502 (2023). 38096285 10.1126/science.adj3502 PMC7616675

[39] Yu, Y., Wang, W. & Zhang, F. The next generation fecal microbiota transplantation: to transplant bacteria or virome. *Adv. Sci.* **10**, e2301097 (2023). 10.1002/advs.202301097 PMC10724401 37914662

[40] Lin, L. et al., The airway microbiome mediates the interaction between environmental exposure and respiratory health in humans. *Nat. Med.* **29**, 1750–1759 (2023). 10.1038/s41591-023-02424-2 37349537

[41] Liu, N. N. et al. Multi-kingdom microbiota analyses identify bacterial-fungal interactions and biomarkers of colorectal cancer across cohorts. *Nat. Microbiol.* **7**, 238–250 (2022). 35087227 10.1038/s41564-021-01030-7 PMC8813618

[42] Wu, X. et al. Gpr35 shapes gut microbial ecology to modulate hepatic steatosis. *Pharm. Res.* **189**, 106690 (2023). 10.1016/j.phrs.2023.106690 36758734

[43] Zhai, L. et al. Gut microbiota-derived tryptamine and phenethylamine impair insulin sensitivity in metabolic syndrome and irritable bowel syndrome. *Nat. Commun.* **14**, 4986 (2023). 37591886 10.1038/s41467-023-40552-y PMC10435514

[44] Devlin, A. S. & Fischbach, M. A. A biosynthetic pathway for a prominent class of microbiota-derived bile acids. *Nat. Chem. Biol.* **11**, 685–690 (2015). 26192599 10.1038/nchembio.1864 PMC4543561

[45] Wang, Y. et al. Sample collection, DNA extraction, and library construction protocols of the human microbiome studies in the International Human Phenome Project. *Phenomics* **3**, 300–308 (2023). 37325707 10.1007/s43657-023-00097-y PMC10260709

[46] Chen, S. et al. fastp: an ultra-fast all-in-one FASTQ preprocessor. *Bioinformatics* **34**, i884–i890 (2018). 30423086 10.1093/bioinformatics/bty560 PMC6129281

[47] Bolyen, E. et al. Reproducible, interactive, scalable and extensible microbiome data science using QIIME 2. *Nat. Biotechnol.* **37**, 852–857 (2019). 31341288 10.1038/s41587-019-0209-9 PMC7015180

[48] Callahan, B. J. et al. DADA2: High-resolution sample inference from Illumina amplicon data. *Nat. Methods* **13**, 581–583 (2016). 27214047 10.1038/nmeth.3869 PMC4927377

[49] Nilsson, R. H. et al. The UNITE database for molecular identification of fungi: handling dark taxa and parallel taxonomic classifications. *Nucleic Acids Res.* **47**, D259–D264 (2019). 30371820 10.1093/nar/gky1022 PMC6324048

[50] Deng, K. et al. Comparison of fecal and blood metabolome reveals inconsistent associations of the gut microbiota with cardiometabolic diseases. *Nat. Commun.* **14**, 571 (2023). 36732517 10.1038/s41467-023-36256-y PMC9894915

[51] Hua, S. et al. Microbial metabolites in chronic heart failure and its common comorbidities. *EMBO Mol. Med.* **15**, e16928 (2023). 37155563 10.15252/emmm.202216928 PMC10245034

[52] Beghini, F. et al. Integrating taxonomic, functional, and strain-level profiling of diverse microbial communities with bioBakery 3. *eLife* **10**, e65088 (2021). 10.7554/eLife.65088 PMC8096432 33944776

[53] Li, D. et al. MEGAHIT v1.0: a fast and scalable metagenome assembler driven by advanced methodologies and community practices. *Methods* **102**, 3–11 (2016). 27012178 10.1016/j.ymeth.2016.02.020

[54] Li, H. Aligning sequence reads, clone sequences and assembly contigs with BWA-MEM. Preprint at https://arxiv.org/abs/1303.3997 (2013).

[55] Kang, D. D. et al. MetaBAT 2: an adaptive binning algorithm for robust and efficient genome reconstruction from metagenome assemblies. *PeerJ* **7**, e7359 (2019). 31388474 10.7717/peerj.7359 PMC6662567

[56] Nissen, J. N. et al. Improved metagenome binning and assembly using deep variational autoencoders. *Nat. Biotechnol.* **39**, 555–560 (2021). 33398153 10.1038/s41587-020-00777-4

[57] Parks, D. H. et al. CheckM: assessing the quality of microbial genomes recovered from isolates, single cells, and metagenomes. *Genome Res.* **25**, 1043–1055 (2015). 25977477 10.1101/gr.186072.114 PMC4484387

[58] Olm, M. R. et al. dRep: a tool for fast and accurate genomic comparisons that enables improved genome recovery from metagenomes through de-replication. *ISME J.* **11**, 2864–2868 (2017). 28742071 10.1038/ismej.2017.126 PMC5702732

[59] Blanco-Miguez, A. et al. Extending and improving metagenomic taxonomic profiling with uncharacterized species using MetaPhlAn 4. *Nat. Biotechnol.* **41**, 1633–1644 (2023). 36823356 10.1038/s41587-023-01688-w PMC10635831

[60] Nayfach, S. et al. CheckV assesses the quality and completeness of metagenome-assembled viral genomes. *Nat. Biotechnol.* **39**, 578–585 (2021). 33349699 10.1038/s41587-020-00774-7 PMC8116208

[61] Kieft, K., Zhou, Z. & Anantharaman, K. VIBRANT: automated recovery, annotation and curation of microbial viruses, and evaluation of viral community function from genomic sequences. *Microbiome* **8**, 90 (2020). 32522236 10.1186/s40168-020-00867-0 PMC7288430

[62] Hyatt, D. et al. Prodigal: prokaryotic gene recognition and translation initiation site identification. *BMC Bioinforma.* **11**, 119 (2010). 10.1186/1471-2105-11-119 PMC2848648 20211023

[63] Aroney, S. T. N. et al. CoverM: read alignment statistics for metagenomics. *Bioinformatics* **41**, btaf147 (2025). 10.1093/bioinformatics/btaf147 PMC11993303 40193404

[64] Russel, J. et al. CRISPRCasTyper: automated identification, annotation, and classification of CRISPR-Cas loci. *CRISPR J.* **3**, 462–469 (2020). 33275853 10.1089/crispr.2020.0059

[65] Jain, C. et al. High throughput ANI analysis of 90K prokaryotic genomes reveals clear species boundaries. *Nat. Commun.* **9**, 5114 (2018). 30504855 10.1038/s41467-018-07641-9 PMC6269478

[66] Li, W. & Godzik, A. Cd-hit: a fast program for clustering and comparing large sets of protein or nucleotide sequences. *Bioinformatics* **22**, 1658–1659 (2006). 16731699 10.1093/bioinformatics/btl158

[67] Csárdi, G. et al. igraph: network analysis and visualization in R. *R package version* **2**, 10–5281 (2024).

[68] Gao, W. et al. Identification and validation of microbial biomarkers from cross-cohort datasets using xMarkerFinder. *Nat. Protoc.* **19**, 2803–2830 (2024). 38745111 10.1038/s41596-024-00999-9

