# Supplementary material for: Multi-kingdom profiling reveals altered gut phage-bacteria-metabolite interactions in MASLD
Source: Nat Commun. 2026 Apr 18;17:5385. doi: 10.1038/s41467-026-71981-0 (PMC13275893; doi:10.1038/s41467-026-71981-0)
Supplement: Supplementary file 1 — Supplementary_information [file 41467_2026_71981_MOESM1_ESM.pdf]

Supplementary Figures

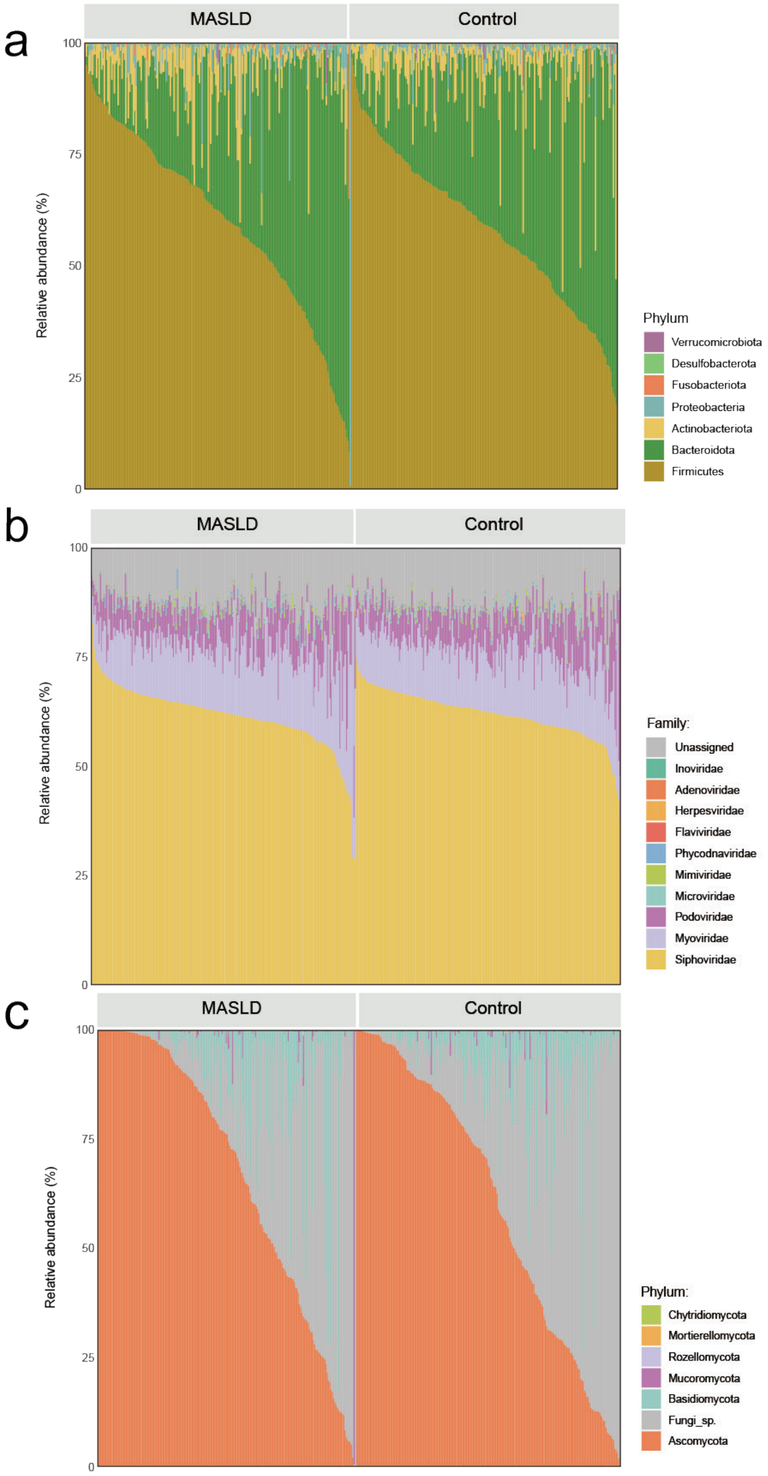

Supplementary Figure 1. Overview of the taxonomic compositions of three gut microbiome kingdoms in our analysis. Phylum-level compositions of bacteria (a, 210 MASLD patients and 210 controls) and fungi (c, 204 MASLD patients and 209 controls), and family-level relative abundance composition of gut virome (b, 210 MASLD patients and 210 controls). Each vertical bar indicates one sample.

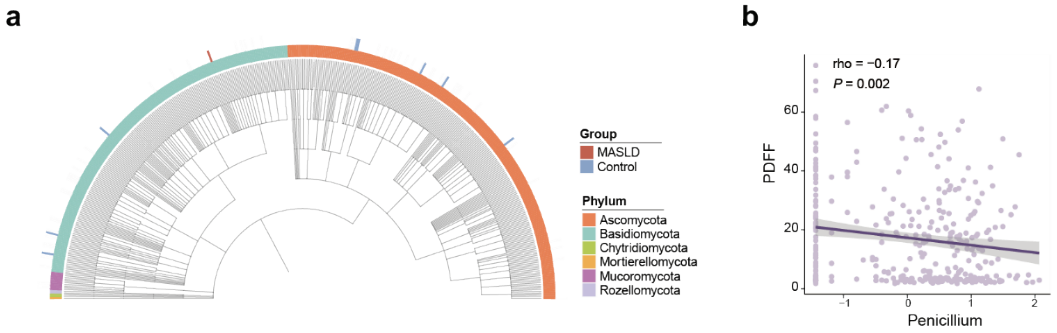

Supplementary Figure 2. Associations of fungal genera with MASLD and its severity. (a) Cladogram of fungal genera based on taxonomic assignments shows fungi significantly altered among MASLD patients (linear regression, FDR < 0.05, 204 MASLD patients vs. 209 controls). (b) Fungal genera *Penicillium* which depleted in MASLD patients is also significantly and inversely associated with PDFF (Spearman rho = -0.17, P = 0.002, n = 354).

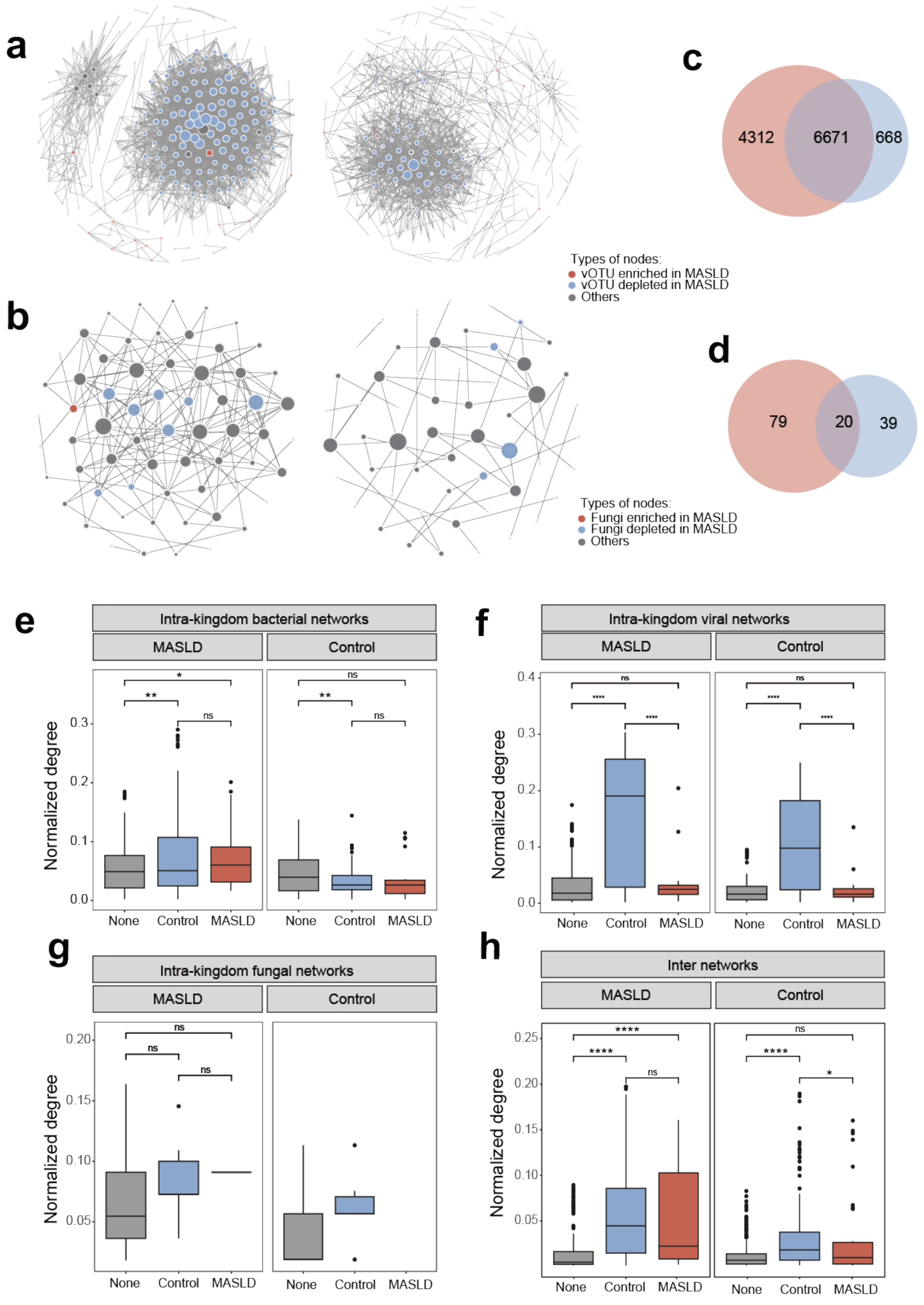

Supplementary Figure 3. Microbial intra- and inter-networks differed between MASLD patients and healthy controls. (a, b) Intra-kingdom networks for viruses (a), and fungi (b) in MASLD patients (left) and healthy controls (right), with network nodes colored by the enriched group and node size indicating network degree. (c, d) Venn diagrams display the number of population-unique and shared correlations for viral

networks (c) and fungal networks (d), chi-square tests are utilized to test whether the numbers of population-specific correlations were statistically different between MASLD and healthy controls (all P values < 0.05). (e, f, g, h) Box plots display node degrees in these networks (e for bacterial, f for viral, g for fungal intra-kingdom networks, h for inter-kingdom networks), with differences assessed using Wilcoxon rank-sum tests (*: $P < 0.05$, **: $P < 0.01$, ***: $P < 0.001$, ****: $P < 0.0001$).

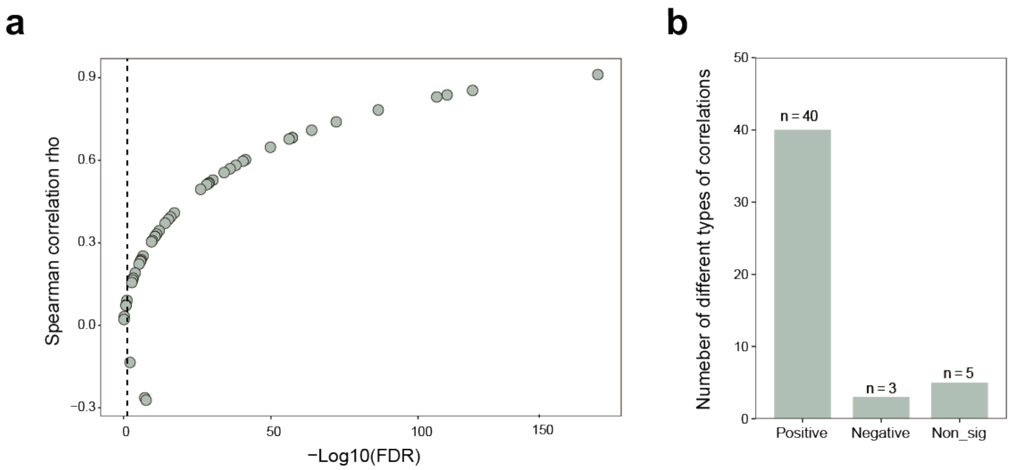

Supplemental Figure 4. Correlations between bacteria species and their corresponding bacteriophages (a) Scatter plot illustrating the Spearman correlations between gut bacteriophages and their bacterial hosts. Each point represents the Spearman correlation for a bacteria species and their bacteriophages and the dotted line indicates the significance threshold at -$\log_{10}(0.05)$. (b) Bar plot categorizing the different types of correlations between the abundance of bacterial species and their bacteriophages, stratified by correlation strength and direction.

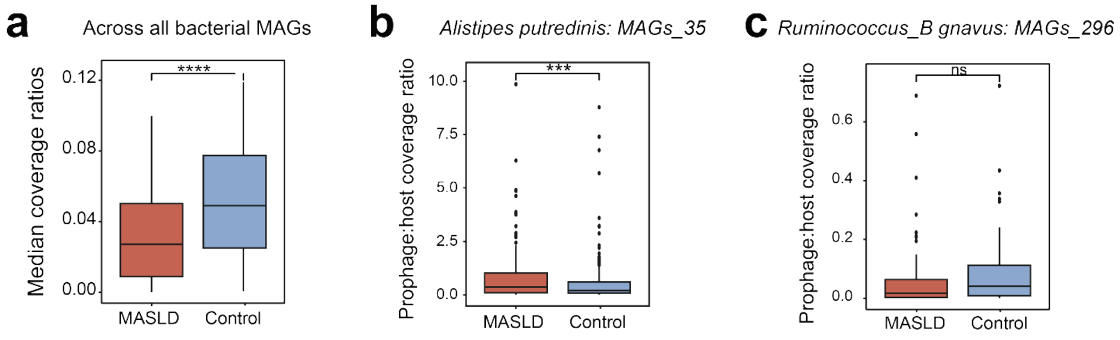

Supplementary Figure 5. Alterations of VBRs for representative bacterial MAGs with MASLD status. (a) Distributions of median ratios of coverages of prophages to their bacterial host regions (n = 420 samples). (b-c) Coverage ratios of prophages in *Alistipes putredinis:* MAGs_35 (b) and *Ruminococcus_B gnavus*: MAGs_296 (c) to their respective host regions. Comparisons are conducted using the two-sided Wilcoxon rank-sum test. Significance levels are indicated as follows: ns $P > 0.05$, *** $P < 0.001$, **** $P < 0.0001$.

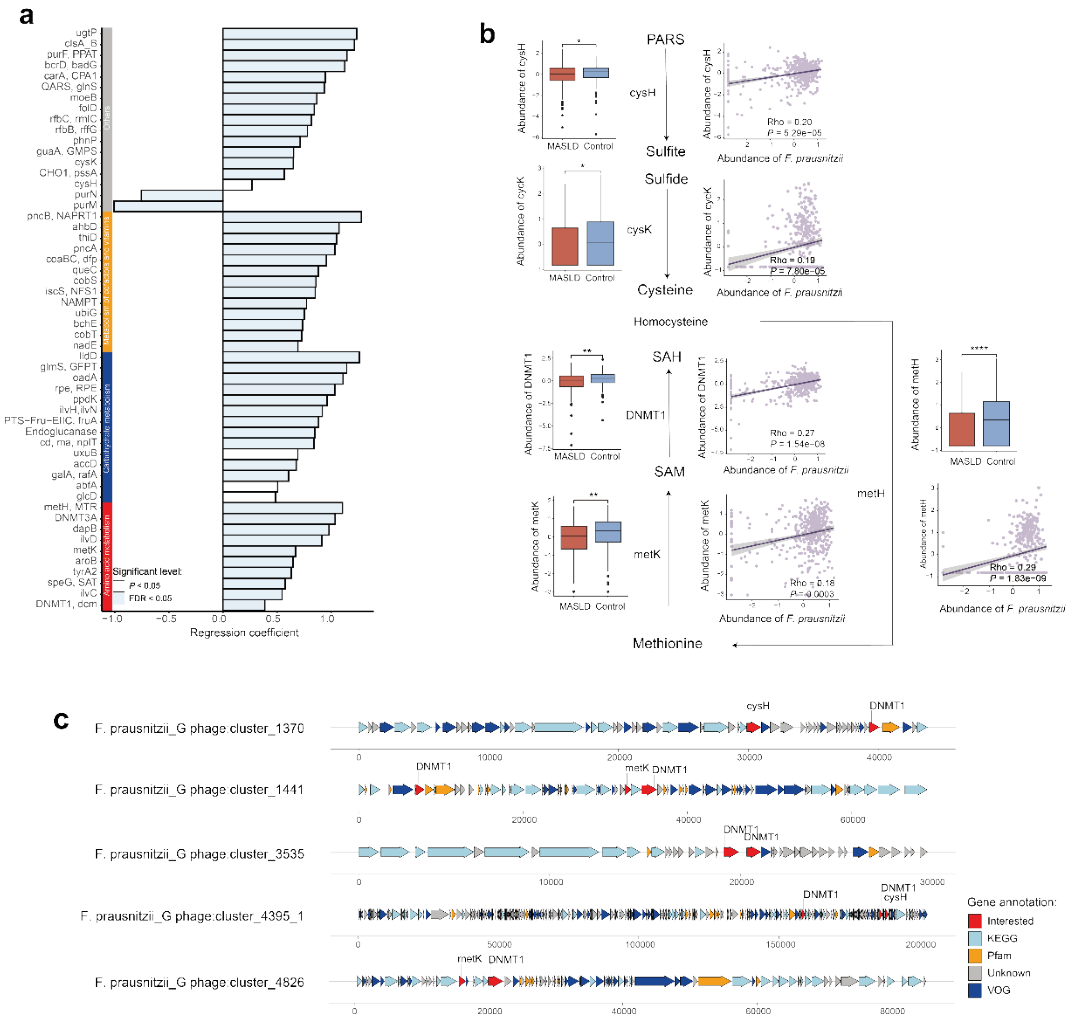

Supplementary Figure 6. AMGs involved in metabolism of Methionine, Homocysteine and Cysteine altered in MASLD patients. (a) Differentially abundant viral auxiliary metabolic genes (AMGs) between MASLD patients and healthy controls. Abundance differences are determined using the linear regression models adjusted for age, gender and BMI. (b) AMGs involved in the metabolism of methionine, homocysteine, and cysteine are identified. Boxplots displayed the differing abundances of these AMGs between groups (MASLD vs. controls). Scatter plots illustrate the correlations between the abundance of specific AMGs and the abundance of *Faecalibacterium prausnitzii*. (c) Genome organisation of five bacteriophages specific to *F. prausnitzii*. The x-axis shows the genome coordinates in bases, with each polygon representing a predicted protein. The colors indicate the source of protein annotation: Pfam (orange), VOG (green), KEGG (light blue). Proteins involved in the metabolism of methionine, homocysteine, and cysteine are highlighted in red, while proteins with

no functional annotations are shown in grey.

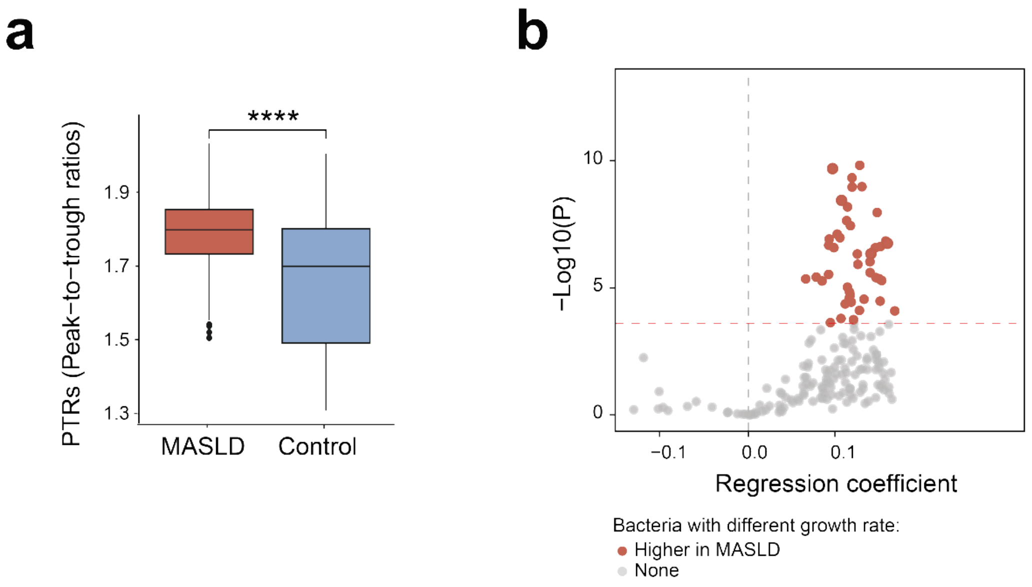

Supplementary Figure 7. Bacterial growth rate differed between MASLD patients and healthy controls. (a) Patients with MASLD show higher gut bacterial growth rate compared to healthy controls (Wilcoxon rank-sum test, ****: $P < 0.0001$). (b) Bacterial MAGs with different growth rate between groups. Bacteria showed higher bacteria growth rate in the MASLD patients are filled in red, and those have comparable growth rate with healthy controls are colored grey.

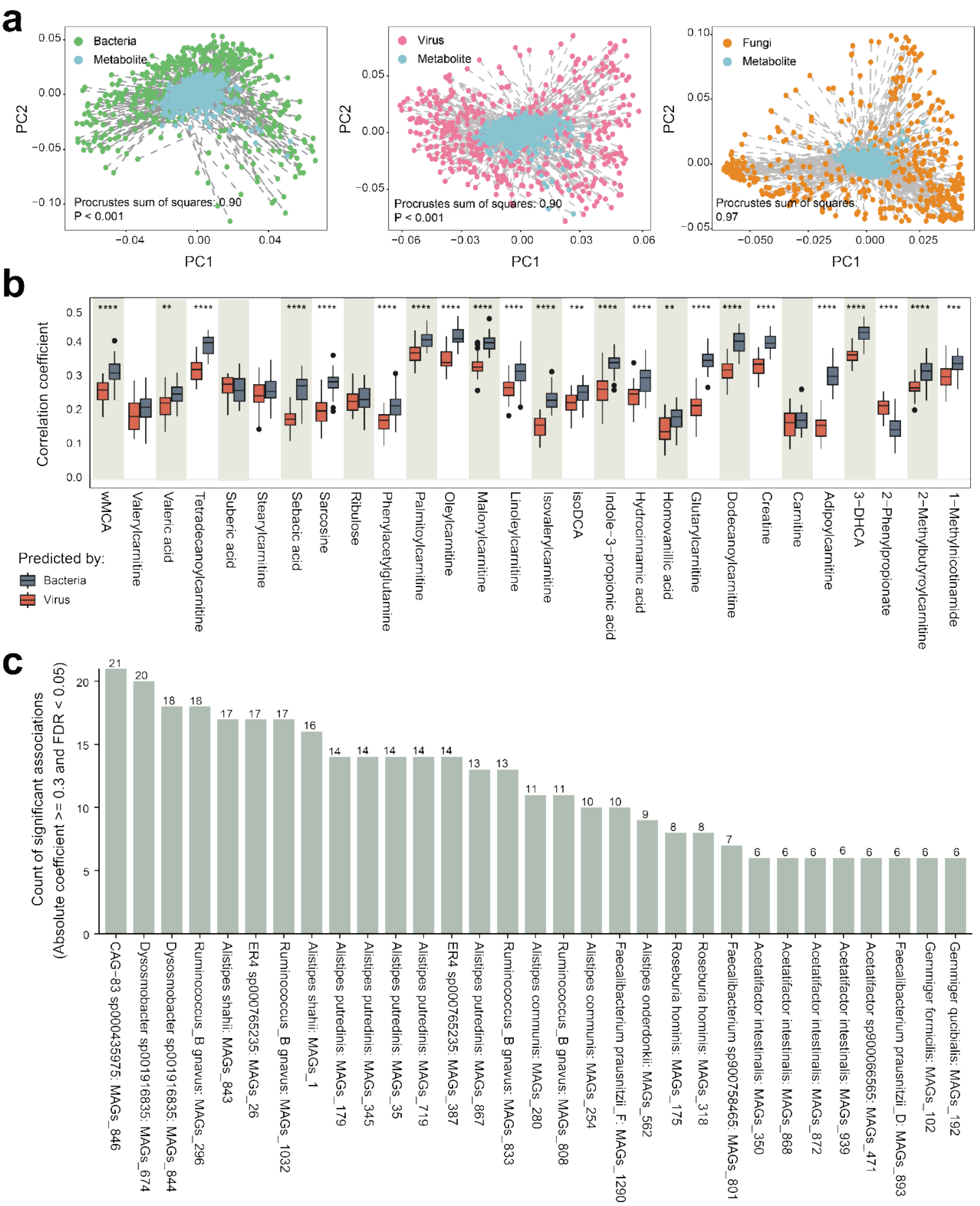

Supplementary Figure 8. Fecal metabolome was closely associated with gut microbiome. (a) Procrustes analysis showing global concordance between gut microbial communities and fecal metabolites (from left to right: gut bacteria, gut viruses, gut fungi versus fecal metabolome). (b) The majority of MASLD-associated fecal metabolites (28 of 39 metabolites) can be well predicted using gut microbial taxonomic profiles. And in most circumstances, predictive power of bacteria taxonomy behaves better than viruses (Wilcoxon rank-sum test, * $P < 0.05$, ** $P < 0.01$, *** $P < 0.001$, **** $P < 0.0001$). (c) Bars exhibit the number of significant associations (defined as

absolute regression coefficient ≥ 0.3 and FDR < 0.05) for each MASLD-associated metabolite with gut bacteria.

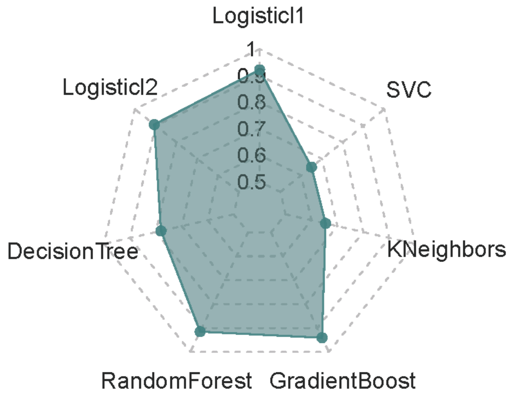

Supplementary Figure 9. Area under the curve of different machine learning algorithms in discriminating MASLD from healthy controls based on gut microbial features. Logisticl1: logistic regression with l1 regularization; Logisticl2: logistic regression with l2 regularization; SVC: support vector classification.

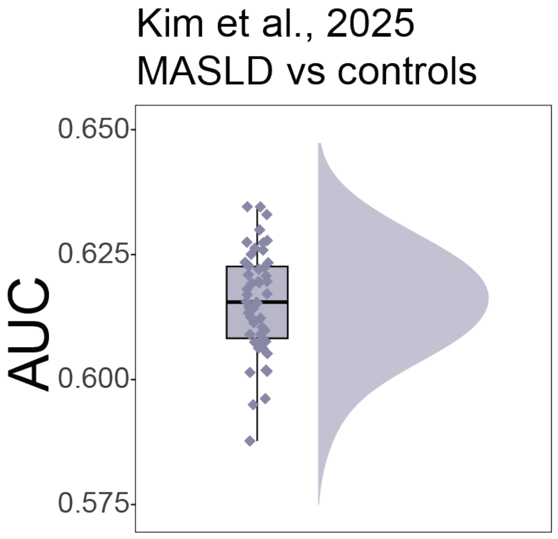

Supplementary Figure 10. Discriminatory power of selected microbial signatures for distinguishing MASLD patients from controls in an external validation cohort.

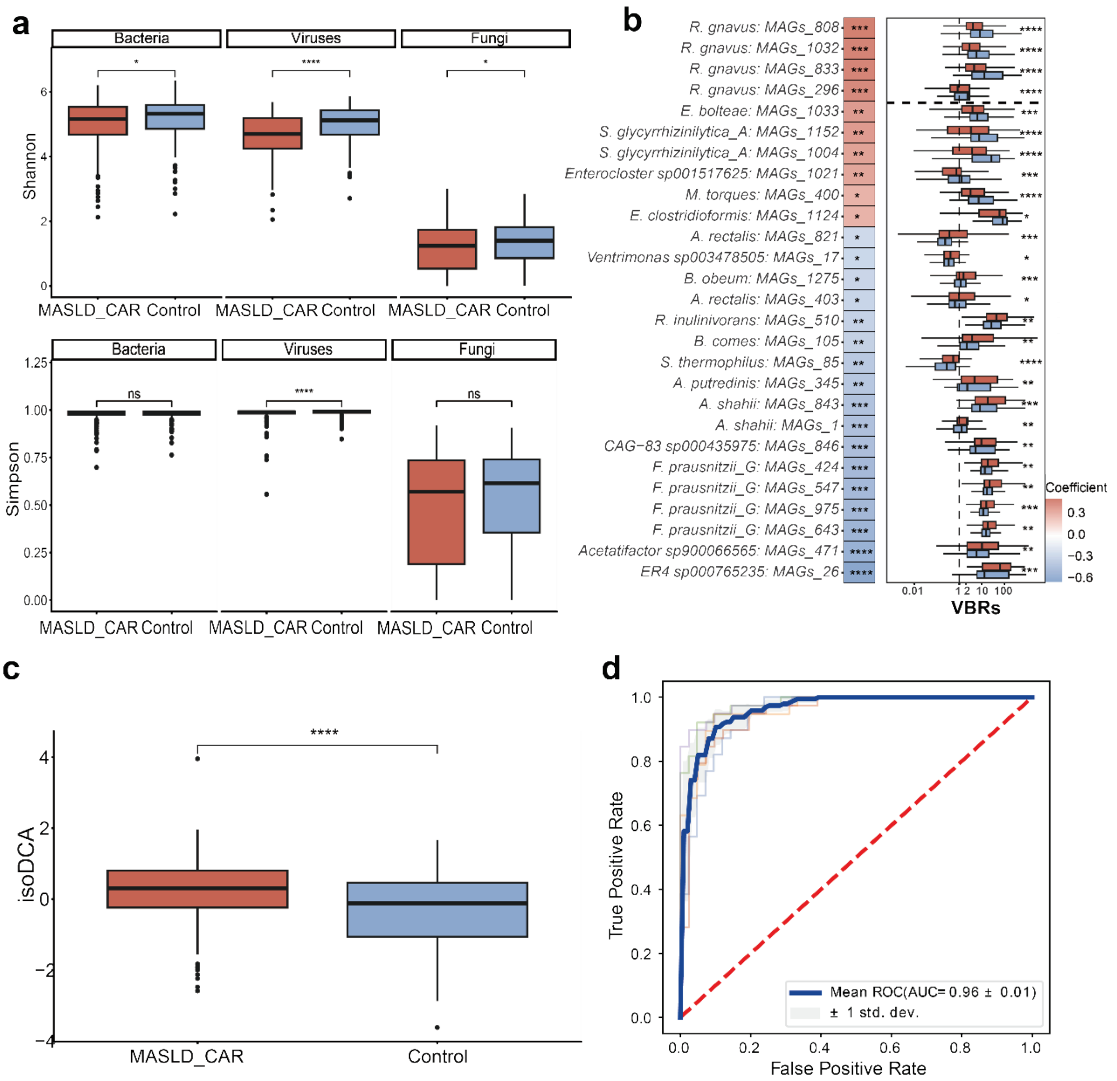

Supplementary Figure 11. Subgroup analysis on MASLD with cardiometabolic risk factors. (a) Alpha diversity metrics (Shannon and Simpson indices) across bacterial, viral, and fungal communities show a significant decrease in MASLD patients with cardiometabolic risk factors (indicated as MASLD_CAR in the figure) compared to healthy controls (Wilcoxon rank-sum test, * P < 0.05, **** P < 0.0001). (b) 27 bacterial MAGs still exhibiting opposite directions in associations between their VBRs and bacterial abundances in relation to MASLD in subgroup analysis (difference of the relative abundance of MAGs were conducted using linear regression with adjustment for age, gender and BMI, and comparisons of VBRs between groups were conducted by Wilcoxon rank-sum test, * $P < 0.05$, ** $P < 0.01$, *** $P < 0.001$, **** $P < 0.0001$). (c) Changes in concentration of isoDCA between healthy controls and MASLD patients with cardiometabolic risk factors (Wilcoxon rank-sum test, **** $P < 0.0001$). (d) ROC curves of performance of classification model based on traditional clinical variables, bacterial and viral signatures.
