# Supplementary material for: Multi-kingdom profiling reveals altered gut phage-bacteria-metabolite interactions in MASLD
Source: Nat Commun. 2026 Apr 18;17:5385. doi: 10.1038/s41467-026-71981-0 (PMC13275893; doi:10.1038/s41467-026-71981-0)
Supplement: Supplementary file 2 — Description of Additional Supplementary Files [file 41467_2026_71981_MOESM2_ESM.pdf]

Description of Additional Supplementary Files

Supplementary Data 1. Clinical and demographic characteristics of the study populations

Supplementary Data 2. Numbers of bacterial MAGs and host-linked vOTUs per species

Supplementary Data 3. Associations of bacterial MAGs with MASLD

Supplementary Data 4. Associations of viral OTUs with MASLD

Supplementary Data 5. Associations of fungal genera with MASLD

Supplementary Data 6. External validation of MASLD-associated bacterial MAGs and vOTUs

Supplementary Data 7. Associations of bacteria growth rate with MASLD

Supplementary Data 8. Annotated fecal metabolites included in the study
