# Supplementary material for: Multi-kingdom profiling reveals altered gut phage-bacteria-metabolite interactions in MASLD
Source: Nat Commun. 2026 Apr 18;17:5385. doi: 10.1038/s41467-026-71981-0 (PMC13275893; doi:10.1038/s41467-026-71981-0)
Supplement: Supplementary file 4 — Reporting Summary [file 41467_2026_71981_MOESM4_ESM.pdf]

nature portfolio

Corresponding author(s): Yan Zheng

Last updated by author(s): Mar 21, 2026

Reporting Summary

Nature Portfolio wishes to improve the reproducibility of the work that we publish. This form provides structure for consistency and transparency in reporting. For further information on Nature Portfolio policies, see our Editorial Policies and the Editorial Policy Checklist.

Statistics

For all statistical analyses, confirm that the following items are present in the figure legend, table legend, main text, or Methods section.

n/a	Confirmed	
☐	☒	The exact sample size (n) for each experimental group/condition, given as a discrete number and unit of measurement
☐	☒	A statement on whether measurements were taken from distinct samples or whether the same sample was measured repeatedly
☐	☒	The statistical test(s) used AND whether they are one- or two-sided<br>*Only common tests should be described solely by name; describe more complex techniques in the Methods section.*
☐	☒	A description of all covariates tested
☐	☒	A description of any assumptions or corrections, such as tests of normality and adjustment for multiple comparisons
☐	☒	A full description of the statistical parameters including central tendency (e.g. means) or other basic estimates (e.g. regression coefficient) AND variation (e.g. standard deviation) or associated estimates of uncertainty (e.g. confidence intervals)
☐	☒	For null hypothesis testing, the test statistic (e.g. F, t, r) with confidence intervals, effect sizes, degrees of freedom and P value noted<br>*Give P values as exact values whenever suitable.*
☒	☐	For Bayesian analysis, information on the choice of priors and Markov chain Monte Carlo settings
☒	☐	For hierarchical and complex designs, identification of the appropriate level for tests and full reporting of outcomes
☐	☒	Estimates of effect sizes (e.g. Cohen's d, Pearson's r), indicating how they were calculated

Our web collection on statistics for biologists contains articles on many of the points above.

Software and code

Policy information about availability of computer code

Data collection	No software was used to collect the data in this study.
Data analysis	Kneaddata (), MegaHIT (v1.2.9), BWA-mem (v0.7.17), MetaBAT2, VAMB (v3.1), CheckM (v1.3.3), dRep (v3.4.0), GTDB-TK (v2.1.0), PHAMB, VIBRANT (v1.2.1), CrisprCasTyper (v1.2.3), Prodigal (v2.6.3), and CoPTR (v1.1.6) were applied to process metagenomic data. R (v4.2.2) was applied to do statistical analysis. xMarkerFinder was utilized to construct the classification model.

For manuscripts utilizing custom algorithms or software that are central to the research but not yet described in published literature, software must be made available to editors and reviewers. We strongly encourage code deposition in a community repository (e.g. GitHub). See the Nature Portfolio guidelines for submitting code & software for further information.

Data

Policy information about availability of data

All manuscripts must include a data availability statement. This statement should provide the following information, where applicable:

- Accession codes, unique identifiers, or web links for publicly available datasets
- A description of any restrictions on data availability
- For clinical datasets or third party data, please ensure that the statement adheres to our policy

Raw metagenomic sequencing data, accompanied by metadata including age, gender, and disease status, have been deposited in the Genome Sequence Archive under accession code PRJCA060134 (https://ngdc.cncb.ac.cn/bioproject/browse/PRJCA060134). Metagenomic sequencing data for external validation datasets were retrieved from the European Nucleotide Archive at the European Bioinformatics Institute (accession numbers: PRJEB6337, PRJEB14215, and PRJNA1246224,

respectively). Raw metabolomic data have been deposited in MetaboLights under accession code MTBLS14073 (https://www.ebi.ac.uk/metabolights/MTBLS14073).

Research involving human participants, their data, or biological material

Policy information about studies with human participants or human data. See also policy information about sex, gender (identity/presentation), and sexual orientation and race, ethnicity and racism.

Reporting on sex and gender	Both males and females were included in the study. Sex was determined based on information recorded on official identification documents. A total of 292 males and 128 females were included. Sex was considered a potential confounder and was adjusted for in association analyses.
Reporting on race, ethnicity, or other socially relevant groupings	All participants included were Han Chinese.
Population characteristics	210 MASLD patients and 210 healthy controls were enrolled. Among the enrolled participants, 30.5% were female, with a median age of 40 years. Details on the characteristics of the participants included in this manuscript were provided in the Supplementary table 1.
Recruitment	Between 2021 and 2022, 210 clinically diagnosed MASLD patients were recruited from Zhongshan Hospital in Shanghai, China. An additional 210 healthy individuals, matched for gender and age (± 5 years), were selected from a healthy cohort recruited at the same hospital during the same period.
Ethics oversight	This study was approved by the Ethics Committee of Zhongshan Hospital (No: B2020-085) and the Research Ethics Committee of the School of Life Sciences at Fudan University (No: FE24199I).

Note that full information on the approval of the study protocol must also be provided in the manuscript.

Field-specific reporting

Please select the one below that is the best fit for your research. If you are not sure, read the appropriate sections before making your selection.

[x] Life sciences [ ] Behavioural & social sciences [ ] Ecological, evolutionary & environmental sciences

For a reference copy of the document with all sections, see nature.com/documents/nr-reporting-summary-flat.pdf

Life sciences study design

All studies must disclose on these points even when the disclosure is negative.

Sample size	Although no formal a priori sample size calculation was performed, the final sample size (n = 420) is comparable to or larger than those of previously published gut microbiome studies in metabolic liver disease and provided sufficient power to detect associations between bacterial, viral, and fungal features and MASLD after adjustment for relevant covariates and multiple testing.
Data exclusions	Participants were excluded if they had significant alcohol consumption (defined as ≥20 grams per day for men and ≥10 grams per day for women); other liver diseases; kidney disorders; cancer or other serious illnesses; had undergone major surgery within the previous eight weeks; or had used antibiotics within the prior four weeks.
Replication	Three independent datasets were used to replicate our findings. And we successfully replicated the our main findings in external populations.
Randomization	No applicable for this study.
Blinding	No applicable for this study.

Reporting for specific materials, systems and methods

We require information from authors about some types of materials, experimental systems and methods used in many studies. Here, indicate whether each material, system or method listed is relevant to your study. If you are not sure if a list item applies to your research, read the appropriate section before selecting a response.

Materials & experimental systems

n/a	Involved in the study
☒	☐ Antibodies
☒	☐ Eukaryotic cell lines
☒	☐ Palaeontology and archaeology
☒	☐ Animals and other organisms
☒	☐ Clinical data
☒	☐ Dual use research of concern
☒	☐ Plants

Methods

n/a	Involved in the study
☒	☐ ChIP-seq
☒	☐ Flow cytometry
☒	☐ MRI-based neuroimaging

Plants

Seed stocks	This study did not involve plants.
Novel plant genotypes	This study did not involve plants.
Authentication	This study did not involve plants.
