# Supplementary material for: Multi-kingdom profiling reveals altered gut phage-bacteria-metabolite interactions in MASLD
Source: Nat Commun. 2026 Apr 18;17:5385. doi: 10.1038/s41467-026-71981-0 (PMC13275893; doi:10.1038/s41467-026-71981-0)
Supplement: Supplementary file 5 — Transparent Peer Review file [file 41467_2026_71981_MOESM5_ESM.pdf]

nature portfolio

Peer Review File

Multi-kingdom profiling reveals altered gut phage-bacteria-metabolite interactions in MASLD

Corresponding Author: Professor Yan Zheng

This file contains all reviewer reports in order by version, followed by all author rebuttals in order by version.

Version 1:

Reviewer comments:

Reviewer #1

(Remarks to the Author)
The authors present a study investigating the dynamics of multi-kingdom interactions in patients with MASLD. They apply shotgun metagenomics and ITS2-based sequencing to measure the fecal bacteriome, mycobiome and viriome, and further correlate these findings with serum metabolome and clinical parameters.

By this, particularly changes in kingdom interactions with Ruminococcus gnavus and Faecalibacterium prausnitzii are placed center stage in the analyses and a machine learning algorithm is developed and benchmared, which is able to identify patients with high accuracy based on the biochemical patient data and multi-kingdom-omics.

While the dynamics and role of the fecal bacteriome is extensively studied and established in the context of MASLD, the findings of alterations in mycobiome and viriome, and there (computational) interaction dynamics are novel to this field.

The manuscript is well written, methodologically sound and presents experiments in an organized fashion. However, I have compiled some comments which I ask the authors to implement.

Comments:

- My major criticism hinges on the number of MAGs of the two most impactful bacterial species F. prausnitzii, and R. gnavus. The conclusions that are shown seem to suggest that these species are over-represented in the MAGs that were generated. If this is the case, then much of the conclusions that are drawn could possibly be influenced by the increased probability of matching in one of the host finding algorithms. A table showing that this is a feeling that does not match the data would be extremely valuable within the main text and should be included. In addition, a normalization that takes into account the number of MAGs per species might also be useful in alleviating this concern. Much of the conclusions are valid given the data that is shown, but the data also seems to consistently show additional MAGs for the organisms that are most impactful. This would reduce the power of the findings as it would be expected the most represented species by number of MAGs would be the host to the most phage. The extrapolation into the metabolic and pathophysiology because of the impact already shown would fall apart if this was just a matter of stochastics.

- The summary of the patient cohort (Supplemental Table S1) suggests that there are patients with hepatic inflammation, thus qualifying for the diagnosis of MASH. It is of relevance to include this discrimination into the analyses. This would allow for higher dynamic resolution of the microbial changes.

- Similarly, the progression of disease, which can be in simplified terms be reduced to increasing fibrosis is not taken in to consideration for most of the analysis with the exception of Figure 2C. If longitudinal clinical data of patients would be availabe, the inter-kingdom connections could be exploited to predict disease progression.

- In addition, the section describing figure 2c (line 165-178) needs rewording. As written, I interpreted “association” in the text to mean a coefficient association by the regression. As I read through it, I think the authors intended to just emphasize that in these datasets these bacteria or phage were found, and thus represent agreement with the associations one sees in the severity indices. If I have this wrong, then there needs additional clarification as both the use of “consistency” within the bacterial MAGs seems to be overstated, and the statement “22 vOTUs, including bacteriophages targeting F. prausnitzii,

were validated in at least one replication dataset.” (line 172) is outright false. Several of the vOTUs including those targeting F. prausnitzii do not have an association in at least one of the replication datasets. As it stands it needs to be reworded.

- Figure 1A (line 105) in the text says “machine learning-based classification models were developed using microbial features to distinguish MASLD patients from healthy controls.” When the authors reflect onto the figure they only have the overview of data that was generated there is no mention of the machine learning tools that were used, and specifically mentioned later on. Simplifying the language here would improve readability.

- Figure 2 A-B: in the figure legend it says “For both trees, phyla, including predicted host phyla for vOTUs, are indicated by different colors.” The legend with the colors is clearly marked, but it’s unclear if there should be an inclusion on the actual trees with the phylum colors. This either needs to be reworded, or corrected on the tree.

- The replication dataset 2 is unclear if any adjustment was made for the linear regression. But the variance between samples is explicitly mentioned both in the results and in the methods. This needs clarification in text as to how this data was treated.

- Where other supervised machine learning algorithms were applied besides X gradient boosting? Do other machine learning algorithms achieve similar accuracy with the same feature sets? The application and comparison of several algorithms, including the fine tuning parameters applied, would further strengthen this part of the

Reviewer #2

(Remarks to the Author)
This study provides a comprehensive multi-kingdom and multi-omics profile of 210 patients with MASLD and matched healthy controls. The methodology was robust and provides a valuable dataset for MASLD microbiome research. The data reveal an enrichment of R. gnavus alongside a reduced virus-to-bacteria ratio for its phages, suggesting a relaxation of phage-mediated control that may favor R. gnavus expansion. This shift coincides with elevated fecal levels of isodeoxycholic acid—a metabolite known to be metabolized by R. gnavus and linked to hepatic steatosis. This rounded association highlights the advantage of this integrated approach. Nevertheless, several aspects warrant careful consideration.

1. The diagnosis of MASLD was based on clinical evaluations and abdominal ultrasonography. This, while understandable, is not ideal. The authors may consider including the presence of at least 1 of 5 cardiometabolic risk factors as a subgroup analysis (Rinella, M. E. et al. 2023).
2. the analysis did not account for dietary intake, which is a dominant driver of gut microbiome composition and a well-established key factor in the pathogenesis of MASLD. The observed microbial and metabolic shifts could be confounded by or secondary to dietary differences between patients and controls, rather than being direct mediators of disease.
3. The abundances of the MAGs were not included in the manuscript. In addition, the metadata related to the uploaded sequencing data is not available. This limits the use of the dataset for others, and for evaluating the validity of their analysis.
4. The authors chose an assembly-based approach for bacterial analysis. This method is robust and suitable for discovering novel MAGs and prophages. However, one of the aims of this study was to develop a non-invasive diagnostic tool. This approach limits generalizability compared to marker gene-based quantification, such as the BioBakery workflow, which is used by the majority of studies focused on microbiome-based classification due to its standardization, speed, and reproducibility.
5. The model relying purely on 4 clinical features achieved an AUC of 0.93, and adding 24 microbial features only improved the AUC by 0.02. This difference looks negligible to me, considering the efforts required for generating the abundance of these features. What the AUC of using only microbial features?
6. In addition, it’s not clear whether clinical features were included in the external datasets. From the description in the manuscript, and the AUCs, one can assume that in dataset from Qin et al., the clinical features were included (AUC>0.9), and for other 2 datasets, only microbial features were used (AUC around 0.7). What is the implication of this classifier?
7. Targeted fecal metabolomics was conducted using the Q300 Metabolite Array Kit, ending up with 212 metabolites. No information was provided for why they chose this panel of relatively limited numbers. What’s the exact class of metabolites that this panel covers, how many of them were produced by microbiome, and how many were primarily host derived.
8. The study would be significantly strengthened by the inclusion of host blood metabolomic data.
9. The growth rate of R. gnavus (MAGs_1032) seems to be significantly higher in MASLD than healthy controls (table S5). The authors may consider mentioning it in the text as it supports their main findings.

Version 2:

Reviewer comments:

Reviewer #1

(Remarks to the Author)
The authors have provided a careful revision which includes both a response to my comments as well as additional analyses. Overall, the manuscript has strongly improved and is now suitable for publication.

Reviewer #2

(Remarks to the Author)
Reviewer #1, Comment 1: The concern that species with greater number of MAGs might be overrepresented. The authors responded that all primary analyses throughout the manuscript are conducted at the individual MAG level rather than aggregated at the species level. This response does not seem very relevant. Although the example of Anaerostipes hadrus (60 MAGs) serves as a counterexample, the authors have not performed a simple normalization that would directly address this concern.

The fact that the model based solely on microbial features using the same gradient boosting framework achieved an AUC of 0.94 in the discovry cohort, should be mentioned in the main text so as readers can compare the model performance across cohort.

I would like to raise another question regarding the external validation: was the discovery-trained model directly applied to external cohorts, or were the same markers used to re-fit a new model in each validation dataset? True external validation requires the former. Re-training tests marker reproducibility, but not model generalizability. Please clarify, and if re-training was done, also report performance of the original model applied directly to external data.

Open Access This Peer Review File is licensed under a Creative Commons Attribution 4.0 International License, which permits use, sharing, adaptation, distribution and reproduction in any medium or format, as long as you give appropriate credit to the original author(s) and the source, provide a link to the Creative Commons license, and indicate if changes were made.
In cases where reviewers are anonymous, credit should be given to 'Anonymous Referee' and the source.
The images or other third party material in this Peer Review File are included in the article’s Creative Commons license, unless indicated otherwise in a credit line to the material. If material is not included in the article’s Creative Commons license and your intended use is not permitted by statutory regulation or exceeds the permitted use, you will need to obtain permission directly from the copyright holder.
To view a copy of this license, visit https://creativecommons.org/licenses/by/4.0/

Reviewer #1 (Remarks to the Authors):

The authors present a study investigating the dynamics of multi-kingdom interactions in patients with MASLD. They apply shotgun metagenomics and ITS2-based sequencing to measure the fecal bacteriome, mycobiome and viriome, and further correlate these findings with serum metabolome and clinical parameters.

By this, particularly changes in kingdom interactions with Ruminococcus gnavus and Faecalibacterium prausnitzii are placed center stage in the analyses and a machine learning algorithm is developed and benchmared, which is able to identify patients with high accuracy based on the biochemical patient data and multi-kingdom-omics.

While the dynamics and role of the fecal bacteriome is extensively studied and established in the context of MASLD, the findings of alterations in mycobiome and viriome, and there (computational) interaction dynamics are novel to this field.

The manuscript is well written, methodologically sound and presents experiments in an organized fashion. However, I have compiled some comments which I ask the authors to implement.

Comments:

- My major criticism hinges on the number of MAGs of the two most impactful bacterial species F. prausnitzii, and R. gnavus. The conclusions that are shown seem to suggest that these species are over-represented in the MAGs that were generated. If this is the case, then much of the conclusions that are drawn could possibly be influenced by the increased probability of matching in one of the host finding algorithms. A table showing that this is a feeling that does not match the data would be extremely valuable within the main text and should be included. In addition, a normalization that takes into account the number of MAGs per species might also be useful in alleviating this concern. Much of the conclusions are valid given the data that is shown, but the data also seems to consistently show additional MAGs for the organisms that are most impactful. This would reduce the power of the findings as it would be expected the most represented species by number of MAGs would be the host to the most phage. The extrapolation into the metabolic and pathophysiology because of the impact already shown would fall apart if this was just a matter of stochastics.

Response:

We thank the reviewer for raising this important methodological concern regarding potential bias arising from unequal numbers of reconstructed MAGs across bacterial species, particularly for *Faecalibacterium prausnitzii* and *Ruminococcus gnavus*. We agree that, if not carefully addressed, species represented by a larger number of MAGs could appear to have increased virus associations simply due to higher representation, thereby inflating host-virus matching probabilities.

To mitigate this concern, we emphasize that all primary analyses throughout the

manuscript are conducted at the individual MAG level rather than aggregated at the species level. This includes associations with MASLD and disease severity indices, bacteria-bacteria interactions, virus-bacterium associations, and correlations with metabolites. Each MAG is therefore treated as an independent analytical unit, and species represented by a greater number of MAGs do not inherently exert greater statistical influence on downstream analyses or conclusions.

The only exception is **Figure 4a**, where bacteriophage-bacterium associations are summarized at the species level, which have been stated on Page 8, Lines 237-238 "*These MAG-level associations were subsequently summarized at the species level for interpretability.*" As now clarified in the revised text and figure legend, this panel is intended solely as a descriptive visualization to illustrate global phage-host interaction patterns and the "Piggyback-the-Winner" phenomenon previously reported in the literature (Knowles *et al.*, *Nature*, 2016; Johansen *et al.*, *Nature Microbiology*, 2023). Importantly, Figure 4a is not used for statistical inference, hypothesis testing, or to support conclusions regarding disease associations, metabolic effects, or pathophysiological mechanisms.

To directly assess whether *F. prausnitzii* and *R. gnavus* are over-represented, we have added a summary table reporting the number of reconstructed MAGs per bacterial species together with the corresponding number of associated viral OTUs (**Supplementary Table 2**, also shown below). As shown in this table, these two species are not disproportionately represented compared with other prevalent taxa in the dataset (four MAGs were constructed for *R. ganvus*, and 12 for four *F. prausnitzii* clades). Notably, the species with the largest number of reconstructed MAGs, *Anaerostipes hadrus* (60 MAGs), does not show significant associations with MASLD, providing a counterexample to the hypothesis that MAG abundance alone drives downstream conclusions.

Together, these clarifications (Page 5, Lines 138-141, Lines 145-150, Lines 154-157; Page 8, Lines 237-238) and additional analyses demonstrate that our conclusions are robust and are not confounded by unequal MAG representation across bacterial species.

Excerpt from Supplemental Table 2. Counts of bacterial MAGs and their targeting vOTUs per species (with selected rows)

Species	MAGs_count	MAGs_included	vOTUs_count	vOTUs_included
s__Anaerostipes hadrus	60	6	98	8
s__Agathobacter faecis	54	43	82	20
s__Bifidobacterium pseudocatenulatum	46	45	57	4
s__Fusicatenibacter saccharivorans	46	46	144	24
s__Ruminococcus_E bromii_B	39	25	56	0
s__Ruminococcus_B gnavus	4	4	32	7
s__Faecalibacterium prausnitzii_D	2	2	40	19
s__Faecalibacterium prausnitzii_E	1	1	14	6
s__Faecalibacterium prausnitzii_F	1	1	1	1
s__Faecalibacterium prausnitzii_G	8	8	128	26

Note: only the top five species with most counts of MAGs constructed and species belonging to *R. gnavus* and *F. prausnitzii* are shown here.

References cited in the authors' response:

Knowles B, Silveira CB, Bailey BA, et al. Lytic to temperate switching of viral communities. Nature. 2016;531(7595):466-470. doi:10.1038/nature17193

Johansen J, Atarashi K, Arai Y, et al. Centenarians have a diverse gut virome with the potential to modulate metabolism and promote healthy lifespan. Nat Microbiol. 2023;8(6):1064-1078. doi:10.1038/s41564-023-01370-6

- The summary of the patient cohort (Supplemental Table S1) suggests that there are patients with hepatic inflammation, thus qualifying for the diagnosis of MASH. It is of relevance to include this discrimination into the analyses. This would allow for higher dynamic resolution of the microbial changes.

Response:

We thank the reviewer for this valuable comment. We agree that distinguishing metabolic dysfunction–associated steatohepatitis (MASH) from non-MASH MASLD is clinically and biologically relevant, as hepatic inflammation represents a key transition along the MASLD disease spectrum and enables higher dynamic resolution of microbiome-associated changes. Based on the presence of hepatic inflammation, we further stratified MASLD patients by explicitly incorporating MASH status into the analyses. Specifically, "*Beyond these indicators, at-risk metabolic dysfunction-associated steatohepatitis (MASH) was defined among MASLD patients using a FibroScan-AST (FAST) score ≥ 0.35 (n = 88)* [24, 25]."(Line 166-168)

This MASH stratification has now been integrated into downstream analyses, including **Figure 2C** "Gut bacterial and viral signatures associated with MASLD", **Figure 4C** "The gut virome dynamics shaped microbial ecology", and **Figure 5B** "Fecal metabolite isoDCA mediates the association of R. gnavus with MASLD". Based on our analysis, inclusion of MASH as a discrete disease category did not alter the main conclusions of the study. Instead, it refined and strengthened the interpretation by revealing consistent and directionally coherent associations of key bacterial MAGs, vOTUs, virus-bacteria interaction metrics and fecal metabolites with inflammatory disease progression.

On Page 6 Lines 176-189: "*Among the 173 MASLD-associated bacterial MAGs, 16 exhibited statistically significant and directionally consistent associations across at least two independent external replication datasets. Similarly, 13 MASLD-associated vOTUs were replicated in at least two external datasets (FDR < 0.05 for the cirrhosis and MASLD replication dataset, P < 0.05 for the steatosis dataset, Figure 2c, Supplementary Table 6). These microbial features further showed consistent pattern across disease progression, showing concordant alterations in MASH patients compared with non-MASH MASLD patients and significant association with liver fat content measured by PDFF (Figure 2c). For example, R. gnavus: MAGs_808 which*

was enriched in MASLD patients in our discovery cohort, was consistently enriched in patients with cirrhosis, sever steatosis, and independent MASLD cohorts, and showed a positive association with liver fat content. In contrast, F. prausnitzii: MAGs_1213, which was depleted in MASLD patients and replicated in external steatosis and MASLD datasets, showed further depletion in MASH patients and was negatively associated with liver fat content."

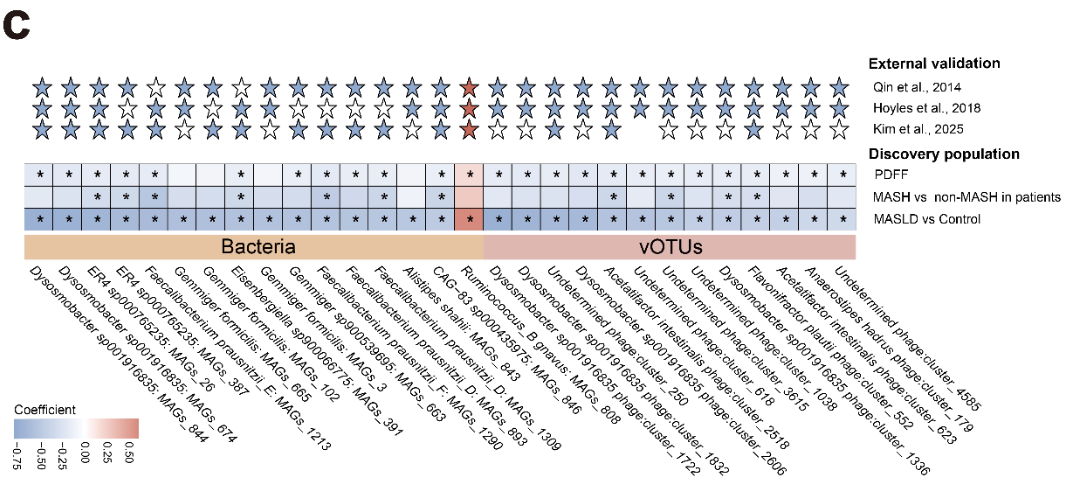

Figure 2. Gut bacterial and viral signatures associated with MASLD. (c) Associations of MASLD-related bacterial MAGs and vOTUs that are validated in at least two external cohorts with MASLD severity. Replication across independent cohorts is indicated by stars, with red and blue denoting positive and negative associations, respectively, between microbiome features and MASLD, hepatic steatosis, or cirrhosis (FDR < 0.05 for Cirrhosis cohort from Qin et al. and MASLD cohort from Kim et al., $P < 0.05$ for the hepatic steatosis cohort from Hoyles et al. due to limited sample size). White stars indicate non-significant associations. Microbial features validated in at least two cohorts are further assessed for their associations with MASLD severity indicators using linear regression adjusted for age, gender, and BMI (* FDR < 0.05 for PDFF and MASLD, $P < 0.05$ for MASH vs. non-MASH).

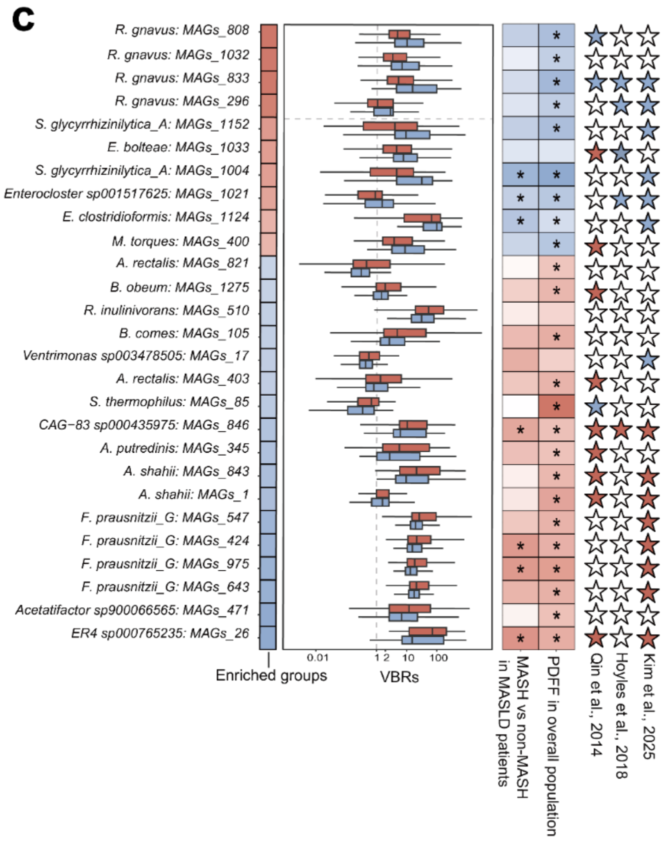

Figure 4. The gut virome dynamics shape microbial ecology. (c) 27 bacterial MAGs exhibiting opposite directions of association between their VBRs and bacterial abundances in relation to MASLD. The VBRs of these MAGs are also linked to disease severity and show consistent alterations in independent validation cohorts. The left heatmap shows differences in bacterial relative abundance between MASLD patients and controls (only taxa with FDR < 0.05 are shown; red indicates enrichment and blue indicates depletion in MASLD). The middle boxplots display differential VBRs for these MAGs between MASLD patients (red) and controls (blue) (FDR < 0.05). The right heatmap denotes comparison of VBRs between non-MASH and MASH patients and associations between bacterial VBRs and liver fat content (*: $P < 0.05$), as well as replications in external cohorts (red and blue indicate positive and negative associations, respectively, with cirrhosis in Qin et al., and MASLD in Kim et al. at FDR < 0.05; and with sever steatosis in Hoyles et al. at $P < 0.05$).

b

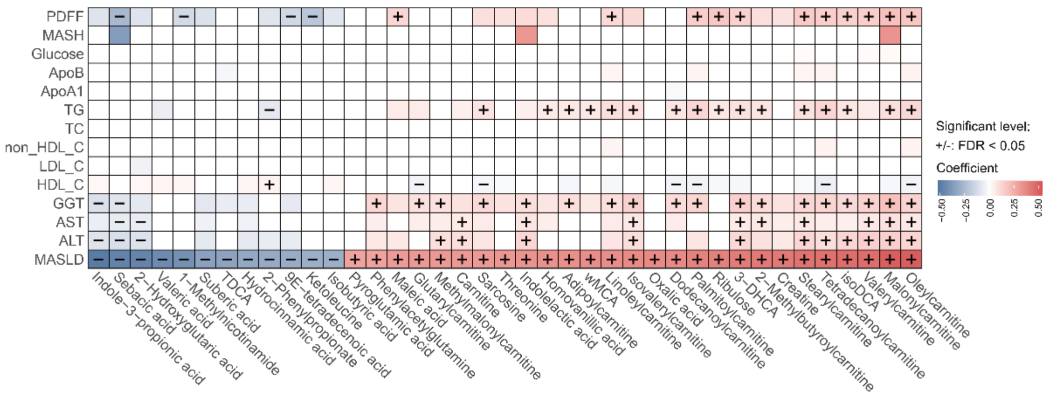

Figure 5. Fecal metabolite isoDCA mediates the association of *R. gnavus* with MASLD. (b) Associations between the MASLD-associated metabolites and clinical traits and disease severity indicators. Cells are colored by direction and significance of association (red: positive with $P < 0.05$, blue: negative with $P < 0.05$, +, -: positive and negative associations respectively with FDR < 0.05).

References cited in the above revised manuscript:

24. Marti-Aguado, D., J.L. Calleja, E. Vilar-Gomez, et al., Low-to-moderate alcohol consumption is associated with increased fibrosis in individuals with metabolic dysfunction-associated steatotic liver disease. J Hepatol, 2024. 81(6): p. 930-940.
25. Newsome, P.N., M. Sasso, J.J. Deeks, et al., FibroScan-AST (FAST) score for the non-invasive identification of patients with non-alcoholic steatohepatitis with significant activity and fibrosis: a prospective derivation and global validation study. Lancet Gastroenterol Hepatol, 2020. 5(4): p. 362-373.

- Similarly, the progression of disease, which can be in simplified terms be reduced to increasing fibrosis is not taken into consideration for most of the analysis with the exception of Figure 2C. If longitudinal clinical data of patients would be available, the inter-kingdom connections could be exploited to predict disease progression.

Response:

We sincerely appreciate the reviewer's constructive advice. We agree that disease progression, which can be approximated in cross-sectional studies by increasing fibrosis severity, represents a critical dimension for understanding microbiome dynamics in MASLD. According to your suggestions, we have included clinical indices implying the progression of disease into our analysis, including associations of severity (PDFF measured using MRI) and clinical MASH status (defined by FibroScan measured CAP, LSM score and AST level) with MASLD-associated microbial features (**Figure 2c**), VBRs of certain bacterial MAGs (**Figure 4c**) and metabolites (**Figure 5b**).

We agree that longitudinal data would provide an even more powerful framework to directly assess temporal dynamics and predictive value of inter-kingdom connections. We have therefore highlighted this point in the Discussion as an important direction for future studies on Page 13 Line 409-411: "*future longitudinal studies in larger, ethnically*

and geographically diverse populations are needed to confirm the generalizability of the proposed phage-bacteria-metabolite axis."

- In addition, the section describing figure 2c (line 165-178) needs rewording. As written, I interpreted "association" in the text to mean a coefficient association by the regression. As I read through it, I think the authors intended to just emphasize that in these datasets these bacteria or phage were found, and thus represent agreement with the associations one sees in the severity indices. If I have this wrong, then there needs additional clarification as both the use of "consistency" within the bacterial MAGs seems to be overstated, and the statement "22 vOTUs, including bacteriophages targeting F. prausnitzii, were validated in at least one replication dataset." (line 172) is outright false. Several of the vOTUs including those targeting F. prausnitzii do not have an association in at least one of the replication datasets. As it stands it needs to be reworded.

Response:

We thank the reviewer for pointing out the ambiguity in our wording and have revised this section to clearly distinguish statistical associations from directional consistency and observational support across cohorts. (Page 6, Line 176-189): "*Among the 173 MASLD-associated bacterial MAGs, 16 exhibited statistically significant and directionally consistent associations across at least two independent external replication datasets. Similarly, 13 MASLD-associated vOTUs were replicated in at least two external datasets (FDR < 0.05 for the cirrhosis and MASLD replication dataset, P < 0.05 for the steatosis dataset, Figure 2c, Supplementary Table 6). These microbial features further showed consistent pattern across disease progression, showing concordant alterations in MASH patients compared with non-MASH MASLD patients and significant association with liver fat content measured by PDFF (Figure 2c). For example, R. gnavus: MAGs_808 which was enriched in MASLD patients in our discovery cohort, was consistently enriched in patients with cirrhosis, sever steatosis, and independent MASLD cohorts, and showed a positive association with liver fat content. In contrast, F. prausnitzii: MAGs_1213, which was depleted in MASLD patients and replicated in external steatosis and MASLD datasets, showed further depletion in MASH patients and was negatively associated with liver fat content.*"

- Figure 1A (line 105) in the text says "machine learning-based classification models were developed using microbial features to distinguish MASLD patients from healthy controls." When the authors reflect onto the figure they only have the overview of data that was generated there is no mention of the machine learning tools that were used, and specifically mentioned later on. Simplifying the language here would improve readability.

Response:

We thank the reviewer for this helpful comment. We agree that the wording in the main text was not fully aligned with the content presented in Figure 1a and may reduce readability. To address this, we have simplified the language in the text describing Figure 1a by removing this sentence, and the revised description can be found on Page

4 Line 105-106 as: "*These datasets were integrated with clinical metadata to identify microbial signatures associated with MASLD (Figure 1a).*"

- Figure 2 A-B: in the figure legend it says "For both trees, phyla, including predicted host phyla for vOTUs, are indicated by different colors." The legend with the colors is clearly marked, but it's unclear if there should be an inclusion on the actual trees with the phylum colors. This either needs to be reworded, or corrected on the tree.

Response:

Thank the reviewer for pointing out this ambiguity. We agree that the original wording of the figure legend could be unclear. We have therefore revised the legend to more precisely describe how phylum-level information is represented in Figure 2A–B, as follow: "*For both trees, phyla, including predicted host phyla for vOTUs, are indicated by the different background shading colors of the tree sectors.*" (Page 30)

- The replication dataset 2 is unclear if any adjustment was made for the linear regression. But the variance between samples is explicitly mentioned both in the results and in the methods. This needs clarification in text as to how this data was treated.

Response:

We thank the reviewer for pointing this out. We have clarified the statistical treatment for replication dataset 2 in the revised method section (Page 20, Line 617-619): "*For dataset 2, no adjustments were applied for this cohort because covariate information in unavailable. Group differences were compared by applying Wilcoxon rank-sum test.*"

- Where other supervised machine learning algorithms were applied besides X gradient boosting? Do other machine learning algorithms achieve similar accuracy with the same feature sets? The application and comparison of several algorithms, including the fine tuning parameters applied, would further strengthen this part of the study.

Response:

We sincerely appreciated the reviewer's advice. In the revised manuscript, we have clarified and expanded the model construction procedure by explicitly separating algorithm evaluation, model selection, and optimization steps. Specifically, we first performed a comparative evaluation of multiple widely used supervised machine learning algorithms, including logistic regression with L1 and L2 regularization, decision tree, random forest, k-nearest neighbors, support vector classification, and gradient boosting. During this initial benchmarking step, all gut microbiome features were included and default model parameters were used to ensure a fair and unbiased comparison across algorithms. Model performance was assessed using cross-validation AUC. This evaluation showed that, while several algorithms achieved robust classification performance, the gradient boosting model consistently yielded the highest accuracy (**Supplementary Figure 9**). Accordingly, gradient boosting was selected as the primary modeling framework for subsequent analyses. Hyperparameters of the gradient boosting model were then optimized using Bayesian optimization with cross-validation AUC as the objective function to construct the final classifier.

We have updated the Results section on Page 10, Lines 305-309 as: "*To develop a robust non-invasive diagnostic classifier for MASLD, we evaluated multiple machine algorithms and selected gradient boosting based on its superior (Supplementary Fig. 9). Using this framework, we constructed a classifier of 28 informative features, including BMI, liver enzymes, and specific gut bacterial and viral taxa, identified by the Boruta feature selection procedure (Figure 6a)*", and Methods section on Pages 20-21, Lines 633-647 as follows: "*To identify an appropriate modeling framework for MASLD classification, we first performed a model evaluation step by benchmarking multiple widely used supervised machine learning algorithms, including logistic regression with L1 and L2 regularization, decision tree, random forest, gradient boosting, k-nearest neighbors, and support vector classification. During this initial evaluation, all available microbial features were included and default model parameters were used to ensure a fair comparison across algorithms. Model performance was assessed using cross-validation AUC. Based on this evaluation, gradient boosting was selected as the primary modeling approach for subsequent analyses. Following model selection, informative microbial features and clinical traits were identified using the Boruta feature selection algorithm. Hyperparameters of the gradient boosting model, including the number of estimator trees, the maximum depth of the trees, the numbers of features per tree, and the maximum samples were then optimized via Bayesian optimization with cross-validation AUC as the objective function to construct the final best-performing classifier.*"

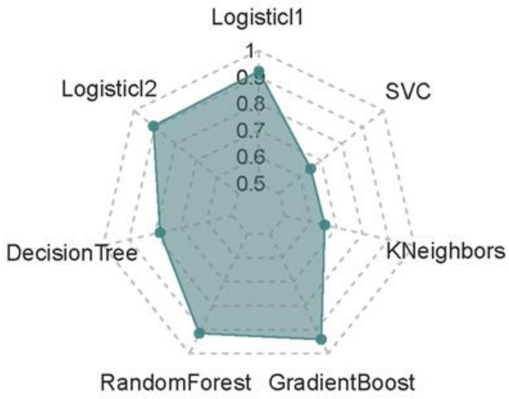

Supplementary Figure 9. Area under the curve of different machine learning algorithms in discriminating MASLD from healthy controls based on gut microbial features. Logisticl1: logistic regression with l1 regularization; Logisticl2: logistic regression with l2 regularization; SVC: support vector classification.

Reviewer #2 (Remarks to the Author):

This study provides a comprehensive multi-kingdom and multi-omics profile of 210 patients with MASLD and matched healthy controls. The methodology was robust and provides a valuable dataset for MASLD microbiome research. The data reveal an enrichment of R. gnavus alongside a reduced virus-to-bacteria ratio for its phages, suggesting a relaxation of phage-mediated control that may favor R. gnavus expansion. This shift coincides with elevated fecal levels of isodeoxycholic acid—a metabolite known to be metabolized by R. gnavus and linked to hepatic steatosis. This rounded association highlights the advantage of this integrated approach. Nevertheless, several aspects warrant careful consideration.

1. The diagnosis of MASLD was based on clinical evaluations and abdominal ultrasonography. This, while understandable, is not ideal. The authors may consider including the presence of at least 1 of 5 cardiometabolic risk factors as a subgroup analysis (Rinella, M. E. et al. 2023).

Response:

We thank the reviewer for this important and thoughtful comment. To further evaluate the validity of our MASLD case definition, we revisited the available clinical metadata for all individuals diagnosed with MASLD. According to the updated consensus proposed by Rinella *et al.* (2023), 196 out of 213 MASLD individuals (92.0%) in this study presented with at least one of the five cardiometabolic risk factors defined in the guideline, indicating that the vast majority of MASLD cases in our study already met the new disease criteria of metabolic dysfunction–associated steatotic liver disease.

Because only a small number of MASLD patients (n = 17) did not meet these criteria, this subgroup was insufficiently powered to support a statistically meaningful comparative subgroup analysis. Nevertheless, to more stringently align our analyses with the updated definition, we restricted downstream analyses to the subset of 196 MASLD individuals with cardiometabolic risk factors (hereafter referred to as MASLD_CAR), and confirmed that the main findings remained consistent. The corresponding results have now been updated in the revised Results section on Page 10 Lines 321-325: "To assess robustness to updated diagnostic criteria, we repeated key analyses in the subset of MASLD patients with ≥1 cardiometabolic risk factor [37](MASLD_CAR, n = 196). The overall patterns of microbiome, virome, and metabolite associations remained highly consistent with the full MASLD cohort, including the key differential taxa, VBR patterns, isoDCA difference, and ROC curve (Supplementary Fig. 11)."

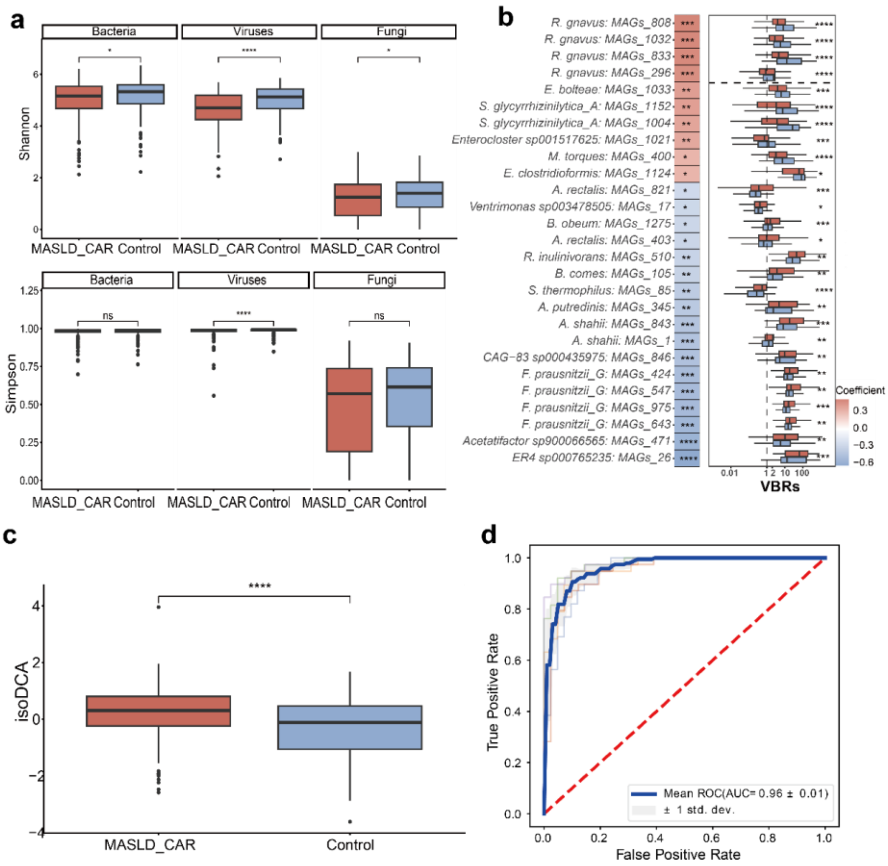

Supplementary Figure 11. Subgroup analysis on MASLD with cardiometabolic risk factors. (a) Alpha diversity metrics (Shannon and Simpson indices) across bacterial, viral, and fungal communities show a significant decrease in MASLD patients with cardiometabolic risk factors (indicated as MASLD_CAR in the figure) compared to healthy controls (Wilcoxon rank-sum test, * $P < 0.05$, **** $P < 0.0001$). (b) 27 bacterial MAGs still exhibiting opposite directions in associations between their VBRs and bacterial abundances in relation to MASLD in subgroup analysis (difference of the relative abundance of MAGs were conducted using linear regression with adjustment for age, gender and BMI, and comparisons of VBRs between groups were conducted by Wilcoxon rank-sum test, * $P < 0.05$, ** $P < 0.01$, *** $P < 0.001$, **** $P < 0.0001$). (c) Changes in concentration of isoDCA between healthy controls and MASLD patients with cardiometabolic risk factors (Wilcoxon rank-sum test, **** $P < 0.0001$). (d) ROC curves of performance of classification model based on traditional clinical variables, bacterial and viral signatures.

Reference cited in the revised manuscript:

37. Rinella, M.E., J.V. Lazarus, V. Ratziu, et al., A multisociety Delphi consensus statement on new fatty liver disease nomenclature. Ann Hepatol, 2024. 29(1): p. 101133.

2. the analysis did not account for dietary intake, which is a dominant driver of gut microbiome composition and a well-established key factor in the pathogenesis of MASLD. The observed microbial and metabolic shifts could be confounded by or secondary to dietary differences between patients and controls, rather than being

direct mediators of disease.

Response:

We thank the reviewer for this important comment. We agree that dietary intake is a dominant determinant of gut microbiome composition and a key factor in MASLD pathogenesis, and that unmeasured dietary differences could influence the observed microbial and metabolic alterations. Detailed dietary information was not systematically collected in this study, which we acknowledge as a limitation. To reduce major confounding, MASLD patients and controls were matched for age and gender, and all analyses were adjusted for BMI, which partially reflects long-term dietary and metabolic status. Nevertheless, residual confounding by diet cannot be excluded. We have explicitly acknowledged this limitation in the Discussion on Page 13, Lines 413-418: "*Although medication use was minimal and analyses were adjusted for age, gender, and BMI, residual confounding, particularly by diet, a dominant determinant of gut microbiome composition and a key driver of MASLD pathogenesis, cannot be excluded, and future studies incorporating standardized dietary assessments and longitudinal designs will be required to disentangle diet-driven effects from disease-associated microbiome alterations and to establish causality.*"

3. The abundances of the MAGs were not included in the manuscript. In addition, the metadata related to the uploaded sequencing data is not available. This limits the use of the dataset for others, and for evaluating the validity of their analysis.

Response:

We thank the reviewer for highlighting the importance of data availability and transparency. We have now clarified and expanded access to both the MAG abundance data and the associated sample metadata. The relative abundances of all reconstructed bacterial MAGs used in this study are provided in Supplementary Table 3, allowing readers to directly evaluate the abundance distributions underlying our analyses. Besides, the metadata associated with the sequencing data, including age, gender, and disease status, have now been made publicly available. Raw metagenomic sequencing data, together with the corresponding metadata, have been deposited in the National Omics Data Encyclopedia under accession number **OEP00005883**.

We have updated the Methods and Data Availability sections accordingly (Page 22, Lines 680-683): "*Raw metagenomic sequencing data, accompanied by metadata including age, gender, and disease status, have been deposited to the National Center for National Omics Data Encyclopedia under accession no. OEP00005883 (https://www.biosino.org/node/project/detail/OEP00005883).*" These updates ensure that all data required to evaluate, reproduce, and reuse our analyses are fully accessible.

4. The authors chose an assembly-based approach for bacterial analysis. This method is robust and suitable for discovering novel MAGs and prophages. However, one of the aims of this study was to develop a non-invasive diagnostic tool. This approach limits generalizability compared to marker gene-based quantification, such as the BioBakery workflow, which is used by the majority of studies focused on microbiome-based classification due to its standardization, speed, and

reproducibility.

Response:

We thank the reviewer for this thoughtful comment and agree that marker gene-based workflows, such as BioBakery, offer clear advantages in standardization, speed, and scalability for microbiome-based diagnostic applications. In this study, however, the assembly-based strategy was intentionally chosen to address a central biological objective: to resolve genome-level bacteria-virus interactions and their potential mechanistic roles in MASLD. This approach enabled us to characterize phage-host relationships and identify a disrupted phage-bacteria-metabolite axis, which would not be accessible using marker-based quantification alone. Moreover, MAG-level resolution captures strain-level variation and mitigates intra-species heterogeneity, allowing more precise identification of microbial features and virus-host interactions relevant to MASLD pathophysiology.

With respect to diagnostic development, we view the assembly-based analyses as a discovery-oriented step aimed at identifying high-confidence microbial and viral markers. These markers can subsequently be translated into more scalable and standardized formats, such as marker gene-based workflows or targeted PCR/sequencing assays, for clinical implementation. We have clarified this distinction between mechanistic discovery and downstream diagnostic translation in the revised manuscript and Discussion on Page 13, Lines 404-406: "*Future studies leveraging marker genes from the selected microbial MAGs and prophages, or targeted qPCR panels, may enable rapid and standardized diagnostic classification.*"

5. The model relying purely on 4 clinical features achieved an AUC of 0.93, and adding 24 microbial features only improved the AUC by 0.02. This difference looks negligible to me, considering the efforts required for generating the abundance of these features. What the AUC of using only microbial features?

Response:

We thank the reviewer for this insightful comment. We agree that the absolute increase in AUC of 0.02 may appear modest. However, it is important to note that the clinical-only model already achieved a high baseline performance (AUC = 0.93), leaving limited room for further improvement. To directly address the reviewer's question, we evaluated a model based solely on microbial features using the same gradient boosting framework, which achieved an AUC of 0.94 (Supplementary Fig. 9), demonstrating that microbial signatures alone carry independent discriminatory information. Importantly, the goal of incorporating microbiome features was not to replace established clinical predictors, but to assess whether they provide complementary information beyond standard risk factors, an incremental effect of similar magnitude that has also been observed in prior studies integrating gut microbiome data into liver disease risk models (e.g., Liu *et al.*, *Cell Metab.* 2022). We therefore view the combined model as a proof-of-concept illustrating the additive, rather than transformative, contribution of microbial features to MASLD risk stratification.

Reference cited in the authors' response:

Liu Y, Méric G, Havulinna AS, et al. Early prediction of incident liver disease using conventional risk factors and gut-microbiome-augmented gradient boosting. Cell Metab. 2022;34(5):719-730.e4. doi:10.1016/j.cmet.2022.03.002

6. In addition, it's not clear whether clinical features were included in the external datasets. From the description in the manuscript, and the AUCs, one can assume that in dataset from Qin et al., the clinical features were included (AUC>0.9), and for other 2 datasets, only microbial features were used (AUC around 0.7). What is the implication of this classifier?

Response:

We thank the reviewer for raising this important point and apologize for the confusion caused by the earlier presentation. We have carefully revisited and corrected the external validation analyses in the revised manuscript. Specifically, we clarify that clinical features were not included in any of the external validation datasets; all reported classification performance in these cohorts was achieved using the selected microbiome-derived features alone in the revised Results section (Lines 312-314): "*Using microbial features only, the classifier achieved a mean AUC of 0.77 in the cirrhosis cohort from Qin et al. (Fig. 6c) and a mean AUC of 0.91 in the steatosis severity cohort from Hoyles et al. (Fig. 6d).* The implication of these results is that the selected microbial features retain discriminatory power across independent populations and disease contexts, supporting the potential utility of the gut microbiome as an independent, non-invasive indicator of MASLD presence and severity, even in the absence of detailed clinical parameters.

7. Targeted fecal metabolomics was conducted using the Q300 Metabolite Array Kit, ending up with 212 metabolites. No information was provided for why they chose this panel of relatively limited numbers. What's the exact class of metabolites that this panel covers, how many of them were produced by microbiome, and how many were primarily host derived.

Response:

We thank the reviewer for this constructive comment regarding our metabolic profiling strategy. The Q300 Metabolite Array Kit was selected as it enables absolute quantification of a focused set of metabolites spanning multiple biologically relevant classes, including bile acids, short-chain fatty acids, amino acids, and organic acids, which are known to play central roles in host-microbiome interactions and metabolic diseases. Compared to untargeted approaches, this targeted panel offers higher analytical sensitivity, quantitative accuracy, and reproducibility across large clinical cohorts, as demonstrated in prior studies using the same platform (e.g., Deng K, *et al.*, *Nat Commun*, 2023; Ning L, *et al.*, *Nat Commun*, 2023; Zhao M, *et al.*, *Cell Metab*, 2024). To improve transparency, we have updated **Supplementary Table 8** to provide detailed annotations for the 211 retained metabolites, including their chemical class and classification as primarily microbially associated or host-derived based on prior literature. These annotations follow the framework described by Hua *et al.* (EMBO Mol

Med, 2023), which also employed the Q300 panel. Corresponding descriptions have been added to the Methods section on Page 17, Lines 507-509: "*These metabolites were annotated as microbially associated or host-specific based on the classification by Hua et al.*[51] *for the Q300 panel (**Supplementary Table 8**).*"

References cited in the authors' response:

Deng K, Xu JJ, Shen L, et al. Comparison of fecal and blood metabolome reveals inconsistent associations of the gut microbiota with cardiometabolic diseases. Nat Commun. 2023;14(1):571. Published 2023 Feb 2. doi:10.1038/s41467-023-36256-y

Ning L, Zhou YL, Sun H, et al. Microbiome and metabolome features in inflammatory bowel disease via multi-omics integration analyses across cohorts. Nat Commun. 2023;14(1):7135. Published 2023 Nov 6. doi:10.1038/s41467-023-42788-0

Zhao M, Ren Z, Zhao A, et al. Gut bacteria-driven homovanillic acid alleviates depression by modulating synaptic integrity. Cell Metab. 2024;36(5):1000-1012.e6. doi:10.1016/j.cmet.2024.03.010

Reference cited in the above revised manuscript:

51. Hua, S., B. Lv, Z. Qiu, et al., Microbial metabolites in chronic heart failure and its common comorbidities. EMBO Mol Med, 2023. 15(6): p. e16928.

8. The study would be significantly strengthened by the inclusion of host blood metabolomic data.

Response:

We thank the reviewer for this valuable suggestion and agree that inclusion of host blood metabolomic profiling would provide complementary insight into systemic metabolic alterations and further strengthen interpretation of host-microbiome interactions in MASLD. In the present study, blood samples were not systematically collected, and blood metabolomic data are therefore not available. Our metabolomic analyses were intentionally focused on fecal samples to directly capture gut microbiome-derived metabolic activity, particularly metabolites central to the bacteria-virus-metabolite interactions investigated here. Systemic metabolic status was partially reflected by available clinical parameters, including BMI and liver-related phenotypes, which were incorporated into the analyses. We have explicitly discussed the complementary value of integrating fecal and blood metabolomics in the revised Discussion (Page 13, Lines 423-427): *"While our metabolomic analyses focused on fecal samples to capture gut microbiome-derived metabolic activity, integration of blood metabolomics in future studies will be essential to link local microbial metabolism with systemic host metabolic responses and MASLD progression."*

9. The growth rate of R. gnavus (MAGs_1032) seems to be significantly higher in MASLD than healthy controls (table S5). The authors may consider mentioning it in the text as it supports their main findings.

Response:

We thank the reviewer for highlighting this supportive observation. We have now incorporated this result into the revised Results section on Page 9 Line 277-279: "*R. gnavus (MAG_1032) exhibits a significantly higher growth rate in MASLD patients compared with healthy controls (Supplementary Table 7), providing additional evidence for its expansion in disease*".

Reviewer #2 (Remarks to the Authors):

Comment 1: The concern that species with greater number of MAGs might be overrepresented. The authors responded that all primary analyses throughout the manuscript are conducted at the individual MAG level rather than aggregated at the species level. This response does not seem very relevant. Although the example of Anaerostipes hadrus (60 MAGs) serves as a counterexample, the authors have not performed a simple normalization that would directly address this concern.

Response:

We thank the reviewer for raising this important point. We agree that unequal number of reconstructed MAGs across species could, in principle, bias MAG-level analyses, and we therefore performed additional species-level sensitivity analyses to test whether this influenced our results. These analyses indicated that the main conclusions were not materially affected by unequal MAG representation across species

Specifically, we performed a species-level normalization analysis by aggregating MAG abundance and VBR values within each species and dividing by the number of MAGs assigned to that species. We then repeated the key association analyses at the species level. The main findings remained qualitatively unchanged, including the significant enrichment of key taxa and the significantly lower VBR of *R. gnavus* in patients with MASLD (see Table R1). These results indicate that the major signals are preserved after normalizing for unequal MAG representation across species.

To further assess whether the observed associations were broadly supported within species rather than driven by only a small subset of MAGs, for each species, we calculated the proportion of MAGs significantly associated with MASLD. As shown in revised Table R1, the major disease-associated signals were generally consistent across MAGs within the same species. Notably, for key species such as *R. gnavus* and *F. prausnitzii*, all reconstructed MAGs showed significant associations with MASLD. Similarly, in VBR-related analyses, all *R. gnavus* MAGs showed significant associations with MASLD.

Together, these results indicate that our conclusions are unlikely to be explained simply by species represented by larger numbers of reconstructed MAGs, and instead reflect broadly consistent species-level signals.

Table R1 Normalized changes of bacterial abundance and VBRs in MASLD patients

Species	Abundant _group	Propotation of MAGs significantly abudant	Higher VBR group	Propotation of MAGs have significantly VBR changes	Species abundant group by normalized abundance	P value by normalized abundance	Higher group by normalized VBRs	P value by normalized VBRs
s__Acetatifactor intestinalis	Control	0.777777778	Control	0.142857143	Control	0.00122238	None	0.991981799
s__Acetatifactor sp900066565	Control	1	MASLD	0.333333333	Control	1.05523E-07	MASLD	4.75462E-07
s__Agathobacter rectalis	Control	1	MASLD	1	Control	0.001137638	MASLD	0.002159157
s__Alistipes putredinis	Control	1	MASLD	0.2	Control	3.47366E-05	MASLD	0.014507772
s__Alistipes shahii	Control	1	MASLD	1	Control	2.95415E-05	MASLD	0.001200357
s__Bariatricus comes	Control	1	MASLD	0.25	Control	0.000784487	MASLD	0.018354103
s__Blautia hansenii	MASLD	1	MASLD	0.5	MASLD	0.000372552	Control	0.014395266
s__Blautia_A obeum	Control	1	MASLD	0.142857143	Control	0.000819182	MASLD	0.003634012
s__CAG-83 sp000435975	Control	1	MASLD	1	Control	0.000149719	MASLD	0.008523492
s__Dysosmobacter sp001916835	Control	1	Control	0.5	Control	1.08193E-08	Control	0.000553629
s__Enterocloster bolteae	MASLD	1	Control	1	MASLD	0.000812209	Control	0.000137649
s__Enterocloster clostridioformis	MASLD	1	Control	1	MASLD	0.008152166	Control	0.003924183
s__Enterocloster sp001517625	MASLD	1	Control	0.5	MASLD	0.001487941	Control	0.00086653

s__ER4 sp000765235	Control	1	MASLD	0.5	Control	2.17789E-07	MASLD	0.000145471
s__Faecalibacterium prausnitzii_D	Control	1	None		Control	2.50813E-05	None	0.45040096
s__Faecalibacterium prausnitzii_E	Control	1	None		Control	5.26641E-07	None	0.72939781
s__Faecalibacterium prausnitzii_F	Control	1	None		Control	1.47061E-05	None	0.42298696
s__Faecalibacterium prausnitzii_G	Control	1	MASLD	0.5	Control	4.27644E-07	MASLD	0.00234577
s__Faecalimonas umbilicata	MASLD	1	MASLD	0.5	MASLD	7.85386E-07	Control	0.00423402
s__Gemmiger formicilis	Control	1	Control	0.333333333	Control	3.69957E-06	None	0.053986332
s__Gemmiger qucibialis	Control	1	Control	0.625	Control	0.000207849	None	0.521682354
s__Mediterraneibacter torques	MASLD	1	Control	1	MASLD	0.011651712	Control	7.61939E-06
s__Roseburia inulinivorans	Control	0.875	Control	0.285714286	Control	0.001047583	None	0.288408661
s__Roseburia inulinivorans	Control	0.875	NAFLD	0.071428571	Control	0.001047583	None	0.288408661
s__Ruminococcus_B gnavus	MASLD	1	Control	1	MASLD	1.8039E-06	Control	4.61162E-08
s__Schaedlerella glycyrrhizinilytica_A	MASLD	1	Control	1	MASLD	0.000427916	Control	4.97818E-11

s__Streptococcus thermophilus	Control	0.5	MASLD	1	Control	0.004592287	MASLD	1.61277E-07
s__Ventrimonas sp003478505	Control	1	MASLD	1	Control	0.002275757	MASLD	0.006819554

The fact that the model based solely on microbial features using the same gradient boosting framework achieved an AUC of 0.94 in the discovery cohort, should be mentioned in the main text so as readers can compare the model performance across cohort.

Response:

We thank the reviewer for this helpful advice; thus, we have added the description in the main text as: "*To develop a robust non-invasive diagnostic classifier for MASLD, we evaluated multiple machine algorithms and selected gradient boosting based on its superior (with an AUC of 0.94, Supplementary Fig. 9)*" on Page 10, lines 303-305.

I would like to raise another question regarding the external validation: was the discovery-trained model directly applied to external cohorts, or were the same markers used to re-fit a new model in each validation dataset? True external validation requires the former. Re-training tests marker reproducibility, but not model generalizability. Please clarify, and if re-training was done, also report performance of the original model applied directly to external data.

Response:

We thank the reviewer for this important point regarding external validation. In this study, our primary objective was to evaluate the cross-cohort reproducibility of the identified microbial markers rather than the direct transferability of the full classifier. Therefore, we re-fitted models in each external dataset using the same set of selected features. We agree that this assesses marker reproducibility, but not strict model generalizability.

As suggested by the reviewer, we applied the original discovery-trained model directly to the external datasets without re-training. The resulting performance was lower (AUC = 0.57 in *Qin et al.*, 0.58 in *Hoyles et al.*, and 0.51 in *Kim et al.*), indicating limited generalizability of the model across datasets. We believe this is largely due to substantial between-cohort heterogeneity, including differences in study design, population characteristics, disease definitions, sequencing and preprocessing pipelines, available features and the absence of fully harmonized clinical covariates. These factors likely affect both feature distributions and model calibration, making direct application of the original model challenging.

By contrast, when models were re-fitted in the external datasets using the same microbial feature set, performance was higher (mean AUC = 0.77 in Qin et al. and mean AUC = 0.91 in Hoyles et al.), supporting the reproducibility of the underlying microbial markers across independent cohorts. We have revised the manuscript accordingly to clearly distinguish marker reproducibility from direct external validation of the original model and to avoid overstating model generalizability. On page 2, lines 49-51: "*A diagnostic classifier integrating bacterial and viral features with clinical parameters distinguished MASLD from controls in our cohort and maintained predictive performance in two external datasets.*" And on Page 10, lines 310-313: "*Using*

microbial features only, we evaluated the reproducibility of the biomarker panel in independent external datasets. The microbial features achieved a mean AUC of 0.77 in the cirrhosis cohort from Qin et al. (Fig. 6c) and a mean AUC of 0.91 in the steatosis severity cohort from Hoyles et al. (Fig. 6d)."
